# Pupillometry tracks cognitive load and salience network activity in a working memory functional magnetic resonance imaging task

**DOI:** 10.1002/hbm.25678

**Published:** 2021-10-08

**Authors:** Julia Fietz, Dorothee Pöhlchen, Florian P. Binder, BeCOME Working Group, Michael Czisch, Philipp G. Sämann, Victor I. Spoormaker

**Affiliations:** ^1^ Department of Translational Research in Psychiatry Max Planck Institute of Psychiatry Munich Germany; ^2^ International Max Planck Research School for Translational Psychiatry (IMPRS‐TP) Max Planck Institute of Psychiatry Munich Germany; ^3^ Max Planck Institute of Psychiatry Munich Germany

**Keywords:** cerebral cortex, gyrus cinguli, humans, magnetic resonance imaging, neuroimaging, pupil, short‐term memory

## Abstract

The diameter of the human pupil tracks working memory processing and is associated with activity in the frontoparietal network. At the same time, recent neuroimaging research has linked human pupil fluctuations to activity in the salience network. In this combined functional magnetic resonance imaging (fMRI)/pupillometry study, we recorded the pupil size of healthy human participants while they performed a blockwise organized working memory task (N‐back) inside an MRI scanner in order to monitor the pupil fluctuations associated neural activity during working memory processing. We first confirmed that mean pupil size closely followed working memory load. Combining this with fMRI data, we focused on blood oxygen level dependent (BOLD) correlates of mean pupil size modeled onto the task blocks as a parametric modulation. Interrogating this modulated task regressor, we were able to retrieve the frontoparietal network. Next, to fully exploit the within‐block dynamics, we divided the blocks into 1 s time bins and filled these with corresponding pupil change values (first‐order derivative of pupil size). We found that pupil change within N‐back blocks was positively correlated with BOLD amplitudes in the areas of the salience network (namely bilateral insula, and anterior cingulate cortex). Taken together, fMRI with simultaneous measurement of pupil parameters constitutes a valuable tool to dissect working memory subprocesses related to both working memory load and salience of the presented stimuli.

## Graphical abstract

The present report is a combined functional magnetic resonance imaging/pupillometry study investigating neural correlates of pupil fluctuations during working memory processing. We showed that pupil size between task conditions with varying working memory load correlates with the frontoparietal network and pupil change within conditions correlates with the salience network. This combined setup allows for a differentiation between working memory subprocesses related to cognitive load and salience of the presented stimuli.

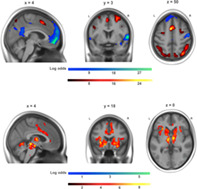

## 1 INTRODUCTION

Working memory is a core executive function (Meule, 2017) responsible for holding information in mind that is actively updated and can be recalled over a short period of time (Baddeley, 1992, 2003). It is a capacity limited system (Luck & Vogel, 1997), confined to the temporary maintenance and manipulation of task‐relevant information, essentially contributing to higher order cognitive functioning and thus to behavior. Although working memory is typically evaluated with behavioral measures, such as reaction times (RTs) and accuracy rates, physiological measures obtained during the actual processing can provide more sensitive and biologically based readouts of individual differences within this cognitive domain (Brückl et al., 2020; Insel et al., 2010).

A substantial body of evidence has shown that pupil diameter increases with cognitive load during working memory performance (Robison & Unsworth, 2019; Unsworth & Robison, 2018; van der Wel & van Steenbergen, 2018; Zokaei, Board, Manohar, & Nobre, 2019), and experimentally high versus low working memory load can be distinguished by pupil diameter with an accuracy of up to 75% (Hogervorst, Brouwer, & van Erp, 2014). Beatty and Kahneman were the first to observe that the pupil dilates as a function of task difficulty and proposed that task‐evoked changes in pupil diameter constitute a reliable physiological index of changes in “processing load” or “mental effort” (Beatty, 1982; Kahneman, Beatty, & Pollack, 1967). To summarize, pupil diameter reflects how cognitive load and attention unfold over time during cognitive processing—presumably reflecting both: task demands and individual processing differences (Alnaes et al., 2014).

In functional magnetic resonance imaging (fMRI) studies, working memory tasks were typically found to activate the frontoparietal network (FPN) across a wide range of experimental paradigms (Owen, McMillan, Laird, & Bullmore, 2005; Rottschy et al., 2012; Wager & Smith, 2003). The lateral prefrontal cortex (PFC) plays a crucial role in working memory, with the rostral‐lateral PFC being related to cognitive processing during a working memory task irrespective of its specific components and the caudal‐lateral PFC being related to working memory load‐dependent effects (Rottschy et al., 2012). Linking physiological readouts of a working memory task with fMRI may elucidate underlying core processes. Moreover, neural correlates of pupil fluctuations in working memory have not been studied yet, particularly not in a joint fMRI/pupillometry setup.

In a single human fMRI/pupillometry study employing neuromelanin‐sensitive imaging, the locus coeruleus (LC) and dorsal anterior cingulate cortex (dACC) were found to correlate with pupil diameter during rest and during performance of an oddball task (Murphy, O'Connell, O'Sullivan, Robertson, & Balsters, 2014). Further neuroimaging work has associated pupil dilation during resting state, fear learning, and reward anticipation with activity in the dACC and bilateral insula (the salience network) (Leuchs, Schneider, Czisch, & Spoormaker, 2017; Schneider et al., 2016; Schneider, Leuchs, Czisch, Sämann, & Spoormaker, 2018). Additionally, a more recent combined fMRI/pupillometry study showed similar results when participants undertook a steady‐state attentional task, revealing a positive correlation of pupil dilation with brainstem, subcortical and cortical regions including the LC, thalamus, posterior cingulate cortex, ACC, and orbitofrontal cortex (DiNuzzo et al., 2019). This line of evidence suggests a link between spontaneous and task evoked (or modulated) pupil dilation and the salience network—sometimes also referred to as the ventral attention network, a system relevant for the detection of behaviorally relevant stimuli and the coordination of neural resources (Menon & Uddin, 2010; Peters, Dunlop, & Downar, 2016).

This implies that, while working memory is associated with FPN activity in the brain (Rottschy et al., 2012), dynamic pupil fluctuations during such processes could reflect the status of salience network involvement. Up to now, pupillometry findings in working memory tasks mainly point to pupil diameter reflecting the cognitive load (Robison & Unsworth, 2019). This notion invites the question of how these two lines of evidence can be integrated. Therefore, this study had two major objectives: first, to examine the neural correlates of varying pupil size as a function of cognitive load in a working memory task, and second, to evaluate the more dynamic pupil fluctuations within a given cognitive load condition and their neural correlates. Disentangling such subprocesses may help us to better understand working memory functioning and thus potential dysfunction in psychiatric disorders (Millan et al., 2012).

To examine this, we recorded the pupil size (equivalent to pupil diameter) of healthy participants while they performed a working memory task inside the MRI scanner, more specifically, the established N‐back task that reliably activates the FPN across participants (Drobyshevsky, Baumann, & Schneider, 2006) and time (Caceres, Hall, Zelaya, Williams, & Mehta, 2009). We hypothesized that pupil size would increase in relation to increasing working memory load. Moreover, in order to investigate the blood oxygen level dependent (BOLD) correlates of working memory related pupil measures, we calculated both pupil size and pupil change (first‐order derivative of pupil size) time courses throughout the block wise organized N‐back task. As previous research could show a relationship between dynamic pupil fluctuations and the salience network (Leuchs et al., 2017; Schneider et al., 2016, 2018), we expected to see similar correlations during the working memory task within each block/condition. In contrast, the cognitive load dependent pupil size differences should manifest itself between the task conditions and should be closer related to the neural correlates of working memory.

## 2 MATERIALS AND METHODS

### 2.1 Participants

One‐hundred and seven participants initially self‐assigned as healthy subjects in the Biological Classification of Mental Disorders (BeCOME) study at the Max Planck Institute of Psychiatry in Munich, Germany (registered on ClinicalTrials.gov: NCT03984084) with measurements obtained up until January 14, 2020 were considered for this analysis (Brückl et al., 2020). The BeCOME study pursues the objective to identify biology‐based classes of affective, anxiety, and stress related mental disorders and it also includes healthy control subjects, following the overall aim of introducing underlying pathophysiological mechanisms into diagnostics and improving translation of biomedical findings into tailored clinical applications.

Exclusion criteria for the BeCOME study in general were any current or past severe medical or neurological conditions, and the current use of psychotropic medication. Anatomical MRI sequences were inspected for incidental brain pathology, or other findings such as large arachnoid cysts that would affect the fMRI analyses. Additionally, all participants took part in the computer‐based Munich Composite International Diagnostic Interview (DIAX/M‐CIDI, Wittchen & Pfister, 1997), which was slightly modified for the BeCOME study through the addition of the assessment of symptoms of depression and anxiety in the past 2 weeks. For the analysis in this current study, we added a post hoc exclusion criterion: full or subthreshold (i.e., one missing symptom) current psychiatric disorder, defined as present within the past 12 months, as verified by the DIAX/M‐CIDI.

Of the overall eligible sample of participants recruited as healthy subjects (*n* = 107, *M*_age_ = 31.6 years, *SD*_age_ = 10.2 years, 73 females), 38 participants were excluded due to the presence of any current full or subthreshold psychiatric disorder, 14 participants were excluded due to pupil (*n* = 10) or fMRI data (*n* = 4) not meeting the below defined quality criteria, two participants were excluded as their pupil data were not recorded due to a technical issue, and one further participant was excluded due to a general technical malfunctioning. After these exclusions, 52 healthy participants (*n* = 52, *M*_age_ = 31.5 years, *SD*_age_ = 9.7 years, 34 females) were included in our analyses. All participants in the remaining sample were non‐smokers and had normal or contact lens corrected vision.

The BeCOME study protocol was in accordance with the Declaration of Helsinki and approved by a local ethics committee (Ludwig Maximilian University of Munich, reference number: 350‐14). All participants provided their written informed consent after the study protocol had been fully explained and were reimbursed for their participation.

### 2.2 The N‐back task

In the N‐back task participants view a sequence of stimuli (e.g., letters) appearing one after another and are asked to respond whenever a current stimulus (= target) matches the one from *n* steps earlier in the sequence. For the N‐back task, we used a set of capital letters as stimuli (consonants B, C, D, G, P, T, W). The task as a whole contained eight blocks, each consisting of 16 stimuli, of the type 0‐back, 1‐back, 2‐back, and fixation. We refrained from adding conditions with higher load due to the design of the BeCOME study (Brückl et al., 2020). Besides healthy participants, patients with psychiatric disorders, for example, mood disorders, were recruited for the BeCOME study in general and cognitive impairments belong to the spectrum of symptoms.

Each condition was presented once in the first half of the task and once in the second (order first half: 0‐back, 2‐back, 1‐back, fixation; order second half: 2‐back, 1‐back, fixation, 0‐back). In the 0‐back condition, participants were instructed to react with a button press when a single prespecified target letter (i.e., W) appeared on the screen. Thus, this control condition had attentional but no working memory demand (i.e., minimal working memory load). In the 1‐back and 2‐back conditions, participants were asked to indicate with a button press whether a letter presented on the screen (= target) matched a letter one or two steps before, respectively. Here, the cognitive load increased with each task condition. All three aforementioned conditions encompassed four target stimuli (25%) with varying letter identities and 12 (75%) non‐target stimuli per block. In the fixation condition, the capital letter X was shown repeatedly as a substitution for the letter stimuli on the screen and no action was required. This condition served as a baseline control as it included a visual input but was lacking a required motor response as well as a working memory and recognition/attentional aspect (Henson, 2007; Zhu et al., 2006).

Before each block, the respective instruction was displayed for 6 s, indicating which condition to follow. The single stimuli, as well as the capital letter X in the fixation condition, were displayed for 500 ms followed by a fixation cross displayed for 2,000 ms before the next stimulus appeared on the screen. In the first 1,000 ms of the fixation, cross display answers were collected.

All stimuli were presented using Presentation Software version 18.01 (Neurobehavioral Systems Inc., Berkeley, CA) in a central position on a monitor located about 2 m behind the end of the scanner bore, which could be seen by the participants via a mirror that was attached to the head coil.

### 2.3 Experimental procedure

The N‐back task was included in the fMRI session on the first BeCOME study day (Brückl et al., 2020). Before performing the task inside the scanner, participants received instructions about the N‐back task in front of a computer outside the scanner by experienced technicians instructed into the BeCOME study.

To ensure that participants fully understood the N‐back task, they completed a short, standardized training of the task outside the scanner room. The training phase involved each condition of the task. After assurance that the task was fully comprehended and any remaining questions were clarified, participants were positioned in the scanner.

### 2.4 Behavior

To compare RTs and accuracy rates between conditions of the N‐back task with varying working memory load, we computed the individual mean RTs and mean accuracy rates across respective trials and conditions. Accuracy was defined as the ratio of pressing the response button in response to targets (= hits) in time, that is, from stimulus onset until maximum 1,000 ms after end of the stimulus presentation in addition to not pressing the response button when non‐targets appeared on the screen (= correct rejections) and the total number of trials. Additionally, we quantified error responses as incorrectly not pressing a button in response to targets (= missed hits) and incorrectly pressing the response button in non‐target trials (= false alarms), see Results section and Figures S1–S4 in the Supplement.

For three participants, the behavioral parameters were not recorded due to technical reasons; therefore, the behavioral analyses were restricted to 49 participants.

### 2.5 Pupillometry

Pupil size and gaze coordinates were recorded with an MR‐compatible eye tracker (EyeLink 1000 Plus; SR Research, Osgoode, ON, Canada), which was placed at the end of the scanner bore and below the presentation monitor, such that the participant's right eye could be tracked via the head coil mirror. Pupil size data were acquired in arbitrary units with a sampling rate of 250 Hz. In order to calibrate the eye gaze position on the monitor, a standard nine‐point calibration procedure was applied. Eye tracking data were processed and analyzed in MATLAB (version 2019b, MathWorks, Natick, MA). Missing data resulting from eye blinks were linearly interpolated between the last saccade before blink onset and the first saccade after blink offset. Saccade markers were provided by EyeLink software (SR Research Ltd.). After this procedure, pupil size data were smoothed by computing the mean of a 200 ms sliding window and z‐transformed to control for variability in average pupil size across participants.

In order to ensure optimal data quality, datasets with more than 20% blink/eye closure‐related missing pupil size values within one block of the task were excluded (*n* = 8), this rate is equivalent to more than 20% of interpolated data within one block. As strong shifts in gaze can interfere with the pupil size detection, we also checked whether the participants' gaze within one block was directed at the center of the screen. For this purpose, we determined the median of the horizontal (x) and vertical (y) gaze data over the course of the task for each participant, yielding a pair of coordinates that indicated the center of the screen on an individual level. Next, we computed the average *SD*s of the x gaze (sd_x = 105.34) and y gaze (sd_y = 91.40) shift across all participants. We defined a cut‐off window by using 3.3 *SD*s around the participant's individual center coordinates. If the participant's gaze remained outside this cut‐off window for more than 20% of the time within one block, the participant was excluded from further analyses (*n* = 2). The procedure of the data quality check was derived from previous literature on pupil fluctuations and their neural correlates in a number of tasks and resting state (Leuchs et al., 2017; Schneider et al., 2016, 2018, 2020). In this study, we adapted the criteria per block (instead of per stimulus) as we were interested in the between and within effects of the blocks, which were also modeled in our subsequent analysis. We also reran the main analyses with including these subjects (for results of this additional analysis see section 2.2 in the Supplement and Figures S5 and S6).

Pupil change was calculated as the first‐order derivative of pupil size. This difference between two consecutive time points of pupil size, equivalent to pupil change, was calculated using MATLAB (version 2019b, MathWorks). For further pupil response quantification, we obtained the pupil maximum value in the search window of 1,000 ms (after stimulus presentation and the light reflex) to 2,500 ms (trial end). From this maximum value, we then subtracted the baseline of the respective trial defined as the mean pupil size between trial onset and 500 ms, which equals the stimulus presentation, just before the light reflex, and after the refractory period of the previous trial.

Additionally, we analyzed a potential tiring effect based on pupil size differences between the first and the second half of the N‐back task, see Results section and Figure S7 in the Supplement.

### 2.6 Statistical analyses of behavioral and pupillometry data

We used Bayesian inferential statistics as implemented in the software package JASP 0.12.2 (https://jasp-stats.org). We performed Bayesian one‐way repeated measures (rm) analysis of variances (ANOVAs) with the N‐back conditions (0‐back, 1‐back, 2‐back, and fixation) as the within subject factor. In a Bayesian repeated measures (rm) ANOVA, different models are compared based on their likelihood given the data. In our case, model comparisons included the null model, stating that there is no effect of condition, and the alternative model with the effect of condition, stating that the conditions differ. The prior probability is equally distributed over those two options (0.5) and the updated probability after observing the data (P(M|data)) provides the relevant output for these analyses. The posterior odds represent the relative plausibility of the alternative model after observing the data, and it is equal to the Bayes factor (BF_10_) multiplied by the prior odds. The Bayes factor quantifies the change of relative plausibility given the data. A BF_10_ of around one indicates that the observed data are equally likely to occur under both models, a BF_10_ between one and three can be interpreted as anecdotal evidence for the alternative hypothesis. A BF_10_ above three but under 10 is seen as moderate evidence for the presence of an effect in favor of the alternative model, and a BF_10_ above 10 is proposed to indicate strong evidence for the presence of an effect. Whereas, for example, a BF_10_ < 1/3, which is mathematically equivalent to BF_01_ > 3, can be interpreted as moderate evidence in favor of the null model (Wagenmakers et al., 2018). For Bayesian ANOVA post hoc tests, Bayesian *t* tests were used. To control for multiple testing, the prior probabilities were adjusted following the Westfall approach (Westfall, 1997). The calculation of the prior model odds depends on the number of respective conditions and in that way each single comparison is considered. The multiplication with the unadjusted Bayes Factor for each pairwise comparison with a Cauchy (0, *r* = 1/sqrt(2)) prior, results in corrected posterior odds (van den Bergh et al., 2020). For reasons of readability, we followed a hybrid approach and also report more commonly used frequentist statistics (Keysers, Gazzola, & Wagenmakers, 2020).

### 2.7 fMRI data acquisition and preprocessing

All participants were scanned in a 3 Tesla MRI Scanner (Discovery MR750, GE, Milwaukee, WI) at the Max Planck Institute of Psychiatry in Munich, Germany. For the data acquisition a 32‐channel head coil, covering 40 slices (AC‐PC orientation of the slices, 96 × 96 matrix, in‐plane field of view 24 × 24 cm^2^, 3 mm slice thickness, 0.5 mm slice gap, resulting voxel size 2.5 × 2.5 × 3.5 mm^3^, echo planar imaging [EPI], TR 2.5 s, TE 30 ms, acceleration factor 2) was used. The N‐back task included a total number of 176 image volumes, of which the first four volumes were discarded to avoid non‐steady‐state effects. Preprocessing and analysis of the fMRI data was performed with MATLAB (version 2019b, MathWorks) using SPM12 (Statistical Parametric Mapping Software, Wellcome Centre for Human Neuroimaging, London, UK, http://www.fil.ion.ucl.ac.uk/SPM), and FSL 6.0 (Wellcome Centre Integrative Neuroimaging, Oxford, UK, https://fsl.fmrib.ox.ac.uk/fsl/fslwiki). Preprocessing of the functional images encompassed—in the order given—(a) realignment using rigid body motion correction with the first image of the task as reference with an additional FSL based rigid body motion to calculate root‐mean‐squared intensity differences between volumes referred to as DVARS, based on Power et al. (2014) and the resulting dummy regressor matrix was saved for later denoising (outliers defined as values larger than 75th percentile plus 1.5 times the interquartile range) (Power et al., 2014); (b) slice time correction considering the bottom‐up acquisition interleaved scheme; (c) coregistration of the time series on a specific single contrast‐rich T2‐weighted EPI image (details in the Supplement); (d) segmentation of this specific image using the unified segmentation algorithm in SPM to separate white matter (WM), gray matter (GM), and cerebrospinal fluid (CSF), (e) spatial normalization entering GM and WM probability maps into the iterative DARTEL algorithm (Ashburner, 2007) using IXI study templates (www.brain-development.org) in MNI space, (f) interpolation to a voxel resolution of 2 × 2 × 2 mm^3^, (g) brain extraction using the FSL brain extraction tool (BET, FSL version 6.0), and (h) spatial smoothing using an isotropic Gaussian Kernel (full width at half maximum 6 × 6 × 6 mm^3^). Denoising was performed including the following set of nuisance covariates in all first level general linear models (GLM): (i) Following the aCompCor strategy (Behzadi, Restom, Liau, & Liu, 2007), five components of WM and CSF (based on segmentation mentioned in Step (d)); (ii) six motion correction coefficients from Step (a) along with their temporal derivatives; and (iii) the DVARS‐based binary matrix. Subjects displaying excessive head movement during scanning—potentially causing motion artifacts—were excluded from the study (*n* = 4). The threshold for exclusion was set at 2 mm translation between two consecutive volumes.

### 2.8 First level analysis

Separate first level GLMs were created for modeling pupil size and pupil change. For analyzing pupil size associated neural activity *between* conditions, we entered the mean pupil size per block (40 s time bins) as a parametric modulation within one blockwise regressor. The blockwise regressor included onset times of all blocks in the four conditions (fixation, 0‐back, 1‐back, 2‐back) presented in the N‐back task.

For analyzing pupil change associated neural activity *within* conditions, we divided the same blocks into 1 s time bins, which constituted the regressor, and entered the corresponding mean pupil change values of those 1 s time bins as its parametric modulation. For this purpose, we downsampled pupil change to 1 Hz. We decided for this approach, as downsampling to the TR (Murphy et al., 2014) before convolution with the hemodynamic response function would result in reduced temporal information, we were particularly interested in.

We used separate models for analyzing neural correlates of pupil size and pupil change in order to prevent collinearity of regressors, which would have a negative effect on statistical power as well as on the parameter estimates (Mumford, Poline, & Poldrack, 2015).

To explicitly model potential effects of condition on pupil change and to avoid the uncontrolled merge with interaction effects, we created an additional first level GLM in which we partitioned the condition regressor (that so far was represented as one single regressor) into four separate regressors (one for 0‐back, one for 1‐back, one for 2‐back, and one for fixation) with onset times of the corresponding 1 s time bins. The equivalent mean pupil change values were entered as parametric modulation.

To examine the neural correlates of trials and their pupil responses, we created another GLM with one regressor encompassing onsets of all trials across the whole task with a duration of 2.5 s. We added the equivalent peak amplitudes in each trial as its parametric modulation.

We used parametric modulators as they provide a flexible analysis approach to disentangle the between and within block/condition effects of the pupil parameters (Leuchs et al., 2017; Schneider et al., 2016, 2018; Wood, Nuerk, Sturm, & Willmes, 2008).

All GLMs were run with nuisance regressors as stated above.

### 2.9 Second level analysis

The group analyses were performed using Bayesian inference as implemented in SPM12. The contrast images of the first level analyses of all participants were used for the model, and tested with Bayesian one‐sample *t* tests against zero (contrasts [+1] and [−1]) for the underlying pupil size, pupil change, and pupil peak GLM. For the statistical maps a minimum effect size of Cohen's *d* = 0.2 and a minimum Bayes factor of ~1,000 was selected (logBF = 3). In cases where relevant separate clusters merged into one larger cluster, the threshold was increased (*d* = 0.5, logBF = 3). For additional analyses, we ran a one‐way ANOVA in SPM12 with one factor (condition) encompassing four levels (fixation, 0‐back, 1‐back, 2‐back; dependent cells) as well as a logical “AND” conjunction analysis to examine to what extent the neural correlates of pupil change depended on condition. All analyses were performed in MATLAB (version 2019b, MathWorks).

The tables detailing the anatomical extent of clusters were created using the automated anatomical labeling (AAL) atlas (AAL 2 toolbox; (Rolls, Joliot, & Tzourio‐Mazoyer, 2015; Tzourio‐Mazoyer et al., 2002). Technically, as the AAL toolbox cannot process posterior probability maps, we used the frequentist maps at voxelwise *p*_FWE_ < .001 threshold for anatomical labeling.

The background image used for depiction of statistical maps (Figures 4–7, S4–S6, S10–S16, S18, and S19 in the Supplement) was generated by unified segmentation of all T1‐weighted images, followed by DARTEL spatial normalization, driven by GM and WM segments (IXI templates, default settings), and application of the flow fields to the bias‐corrected whole head image. Supplemental Figure S8 illustrates the spatial normalization procedure and compares the matching of the resulting normalized functional images with the template space.

## 3 RESULTS

### 3.1 Behavioral results

**FIGURE 1:**
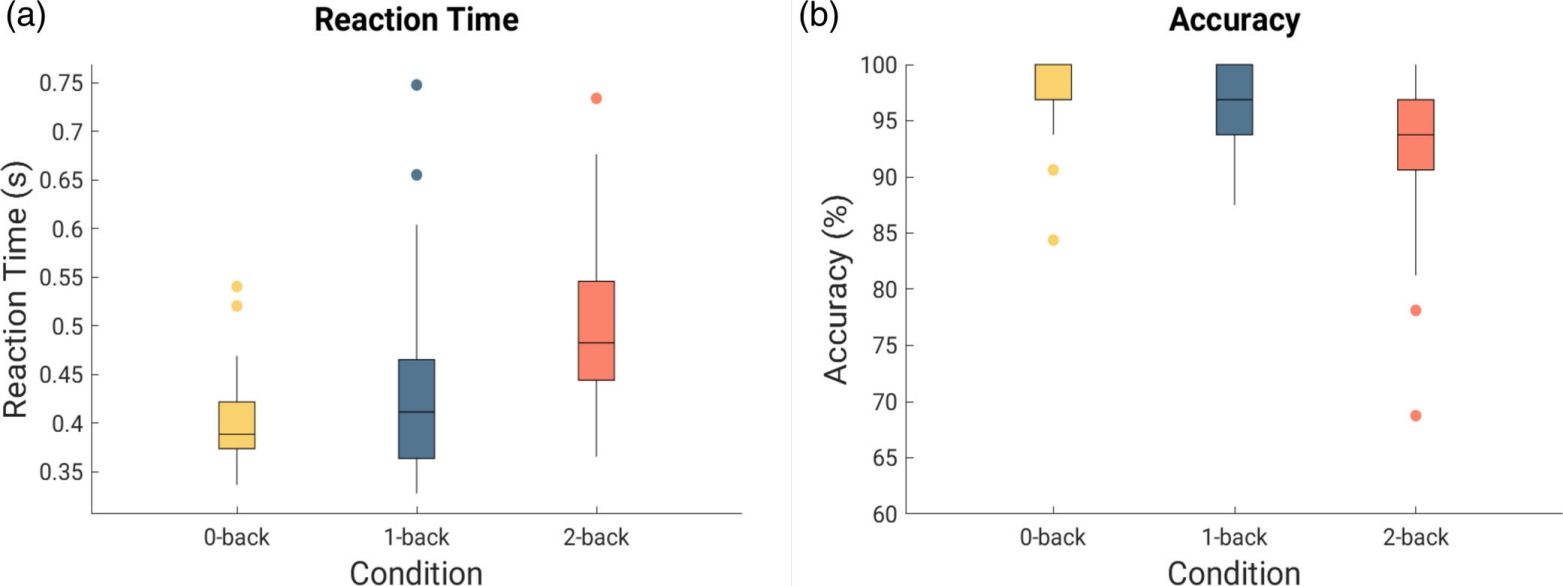
Boxplots showing (a) reaction time (RT) and (b) accuracy in the N‐back task in each condition. Horizontal line within each box denotes median values; boxes extend from the 25th to 75th percentile; vertical extending lines (whisker) denote values outside the interquartile range (IQR). The upper whisker extends to the largest value no further than 1.5 × IQR and the lower whisker extends to the smallest value no further than 1.5 × IQR; dots beyond the end of the whiskers represent outliers

The Bayesian one‐way rmANOVA yielded very strong evidence for an effect of condition on RT with P(M|data) = 1.0, BF_10_ = 2 × 10^11^, *F*_(2,96)_ = 43.1, *p* < .001 (Figure 1a). Descriptive statistics are listed in Table 1. This shows that RT depended on the working memory load level of the respective condition in the N‐back task. The adjusted posterior odds show (a) strong evidence that 0‐back differed from 1‐back (odds of 15), (b) very strong evidence that 0‐back differed from 2‐back (odds of 4 × 10^10^), and (c) very strong evidence that 1‐back differed from 2‐back (odds of 1.7 × 10^3^). Results of the Bayesian post hoc tests are listed in Table S1 in the Supplement.

**TABLE 1:** Descriptive statistics for RT

Condition	Mean	*SD*	*N*	95% credible interval	
				Lower	Upper
0‐back	0.400	0.045	49	0.386	0.413
1‐back	0.432	0.087	49	0.407	0.457
2‐back	0.498	0.082	49	0.474	0.521

*Note*: RT is presented in s.

Abbreviation: RT, reaction time.

Regarding accuracy, the Bayesian one‐way rmANOVA showed very strong evidence for an effect of condition on accuracy with P(M|data) = 1.0, BF_10_ = 1 × 10^6^, *F*_(2,96)_ = 21.4, *p* < .001 (Figure 1b). Descriptive statistics for accuracy are depicted in Table 2. The adjusted posterior odds show (a) very strong evidence that 0‐back differed from 2‐back (odds of 7.4 × 10^3^), (b) very strong evidence that 1‐back differed from 2‐back (odds of 2.4 × 10^2^), and (c) some evidence for no differences between 0‐back and 1‐back (odds of 0.3). Results of the Bayesian post hoc tests are listed in Table S2 in the Supplement.

**TABLE 2:** Descriptive statistics for accuracy

Condition	Mean	*SD*	*N*	95% credible interval	
				Lower	Upper
0‐back	0.980	0.037	49	0.969	0.990
1‐back	0.970	0.034	49	0.960	0.980
2‐back	0.929	0.066	49	0.910	0.948

### 3.2 Pupillometry

**FIGURE 2:**
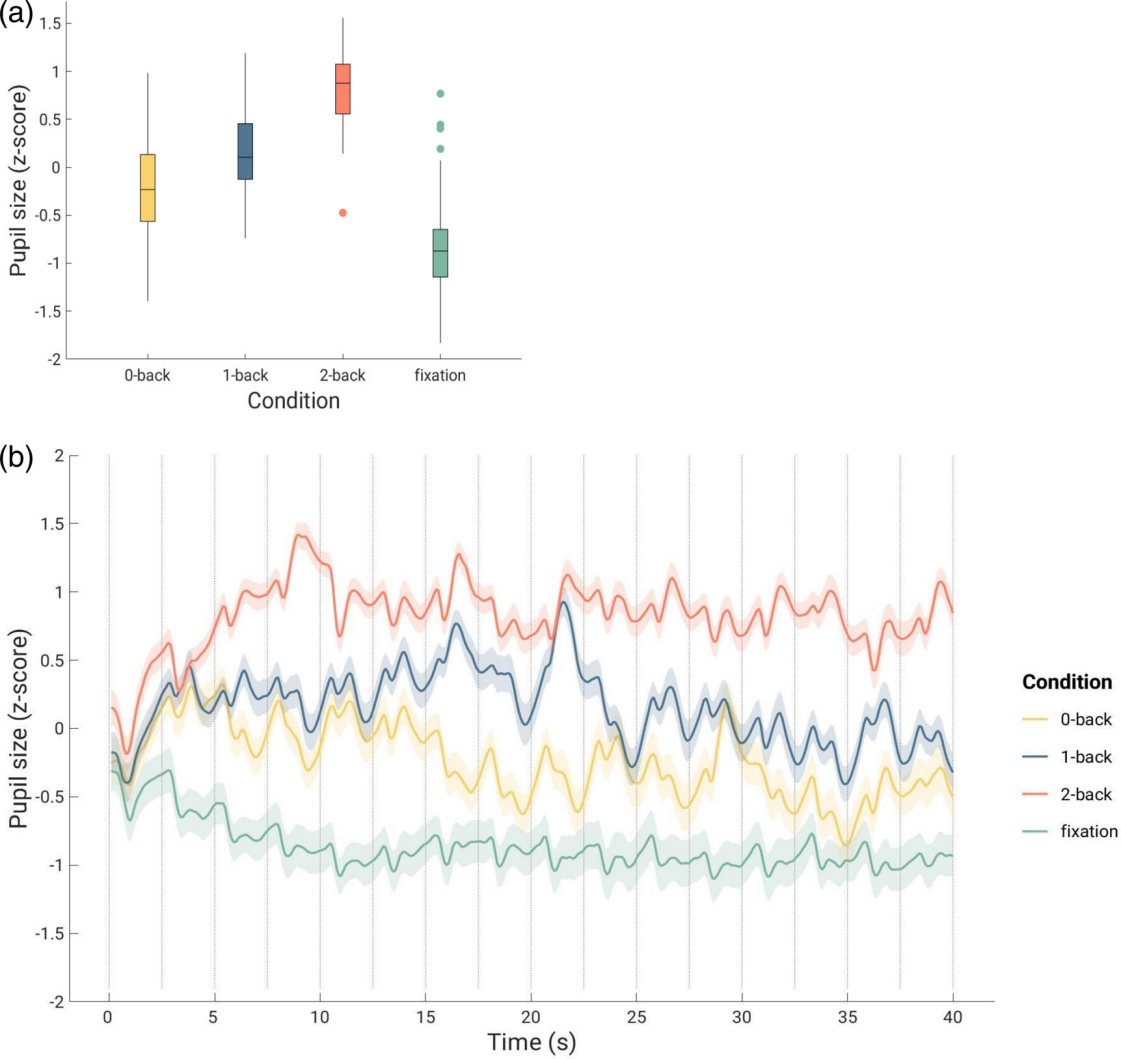
(a) Boxplot showing the distribution of pupil size calculated as the mean of pupil size values in each condition (one value per participant). (b) Mean pupil size over the time course of a task block within each condition. The x‐axis represents the length (40 s) of one block. We downsampled the pupil size values to 10 Hz and calculated the mean of both halves of the task within each condition. The shaded area represents 95% confidence intervals of the mean. The gray vertical lines indicate trial onsets. The last gray vertical line at 40 s indicates the end of the block

For pupil size, the Bayesian one‐way rmANOVA yielded very strong evidence for an effect of condition on pupil size P(M|data) = 1.0, BF_10_ = 1 × 10^71^, *F*_(2,102)_ = 166.0, *p* < .001, indicating that pupil size depended on working memory load (Figure 2). The adjusted posterior odds show (a) very strong evidence that 0‐back differed from 1‐back (odds of 1.6 × 10^5^), 2‐back (odds of 1 × 10^20^), and fixation (odds of 9 × 10^7^); (b) very strong evidence that 1‐back differed from 2‐back (odds of 1 × 10^13^) and fixation (odds of 4 × 10^16^); and (c) very strong evidence that 2‐back differed from fixation (odds of 1 × 10^27^). The results for the post hoc tests are shown in Table S3 in the Supplement.

**FIGURE 3:**
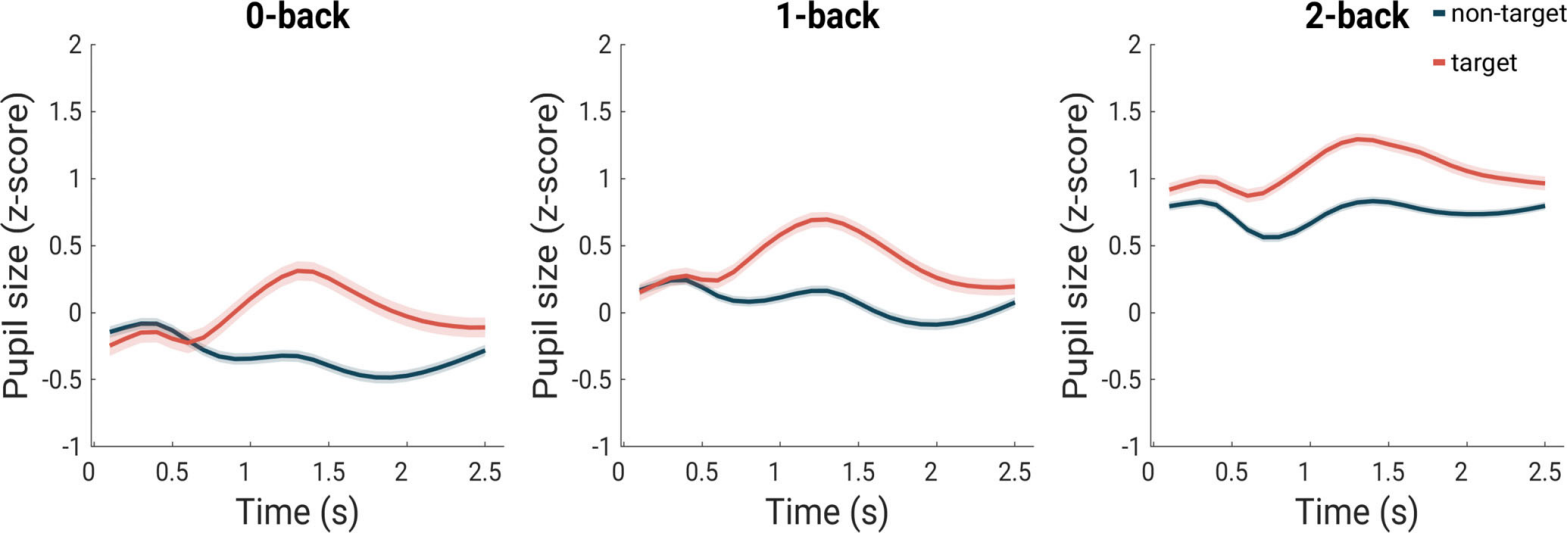
Mean pupil size in response to non‐target (in blue) and target (in red) trials in the three active N‐back task conditions (0‐back, 1‐back, and 2‐back). The x‐axis represents the length (2.5 s) of one trial. Between 0 and 0.5 s, the stimulus is presented, between 0.5 and 1.5 s, the response, if necessary, is collected and between 1.5 and 2.5 s is the inter trial interval. The shaded area represents 95% confidence intervals of the mean

To investigate event‐related pupil responses, we analyzed target and non‐target trials in the three active N‐back task conditions (0‐back, 1‐back, and 2‐back). It becomes evident that the pupil shows a stronger dilation in relation to target trials compared to non‐target trials in all three conditions (Figures 3 and S9 in the Supplement). To examine differential scores, we subtracted peak values of the non‐target trials from the peak values of the target trials for each condition (0‐back, 1‐back, and 2‐back). A Bayesian one‐way rmANOVA revealed strong evidence for an effect of condition, P(M|data) = 1.0, BF_10_ = 2.94 × 10^11^, *F*_(1.73,88.19)_ = 41.73, *p* < .001 (Greenhouse–Geisser corrected as assumption of sphericity was violated according to Mauchly's test). The adjusted posterior odds show (a) very strong evidence that the differential peak amplitude in the 0‐back condition (*M* = 0.59, *SD* = 0.3) was larger than the values for the 1‐back (*M* = 0.42, *SD* = 0.22, odds of 63.79) and 2‐back conditions (*M* = 0.23, *SD* = 0.22, odds of 1.89 × 10^8^), and (b) very strong evidence that the values for the 1‐back condition were larger than the vales for the 2‐back condition (odds of 5.72 × 10^3^).The descriptive statistics of pupil peaks (maximum pupil amplitude minus baseline) are provided in Table 3.

**TABLE 3:** Descriptive statistics of pupil peaks

	0‐back T	0‐back NT	1‐back T	1‐back NT	2‐back T	2‐back NT
Mean	0.72	0.13	0.66	0.26	0.57	0.34
*SD*	0.36	0.13	0.33	0.21	0.33	0.22
Minimum	0.15	−0.13	0.13	−0.16	−0.04	0.04
Maximum	1.78	0.44	1.50	0.79	1.47	1.07

*Note*: T = target trials, NT = non‐target trials, *SD* = standard deviation.

Furthermore, we analyzed the relation between pupil size and performance on a trial‐by‐trial basis. We first down‐sampled the pupil size vector to a resolution of 10 Hz in each trial and then took the mean of the pupil size values per trial (duration of 2.5 s) of all target trials in the three active N‐back conditions (0‐back, 1‐back, and 2‐back) with correct responses (= hits). Then, we calculated the Pearson correlation coefficient between these trial‐wise pupil size values and its respective RTs, which yielded a weak but significant (positive) correlation, *r* = .23, *p* < .001.

### 3.3 Functional magnetic resonance imaging

#### 3.3.1 Neural activity related to pupil size between conditions

**FIGURE 4:**
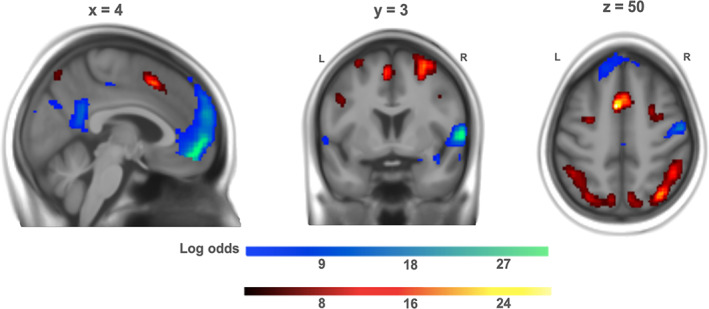
Neural correlates of pupil size between conditions. Hot colors: blood oxygen level dependent (BOLD) activity positively correlated with pupil size. Cold colors: BOLD activity negatively correlated with pupil size (*d* = 0.2, logBF >3). L = left, R = right

The second level GLM of the mean pupil size values per block revealed very strong evidence for positively correlated BOLD activity mainly in the FPN: the dorsolateral PFC (superior frontal gyrus, middle frontal gyrus, supplementary motor area [SMA]), ventrolateral PFC (inferior frontal gyrus), posterior parietal lobules (angular gyrus), in addition to activity in the bilateral insula, *d* = 0.2, logBF >3 (Figure 4). The reverse contrast revealed very strong evidence for negatively correlated BOLD activity in bilateral clusters of the precuneus, orbitofrontal gyrus, as well as activation in the anterior cingulate gyrus, posterior cingulate gyrus, and the lateral parietal cortex (precentral and postcentral gyrus), *d* = 0.2, logBF >3. For a detailed listing of these clusters, see Table S4 in the Supplement.

#### 3.3.2 Neural activity related to pupil change within conditions

**FIGURE 5:**
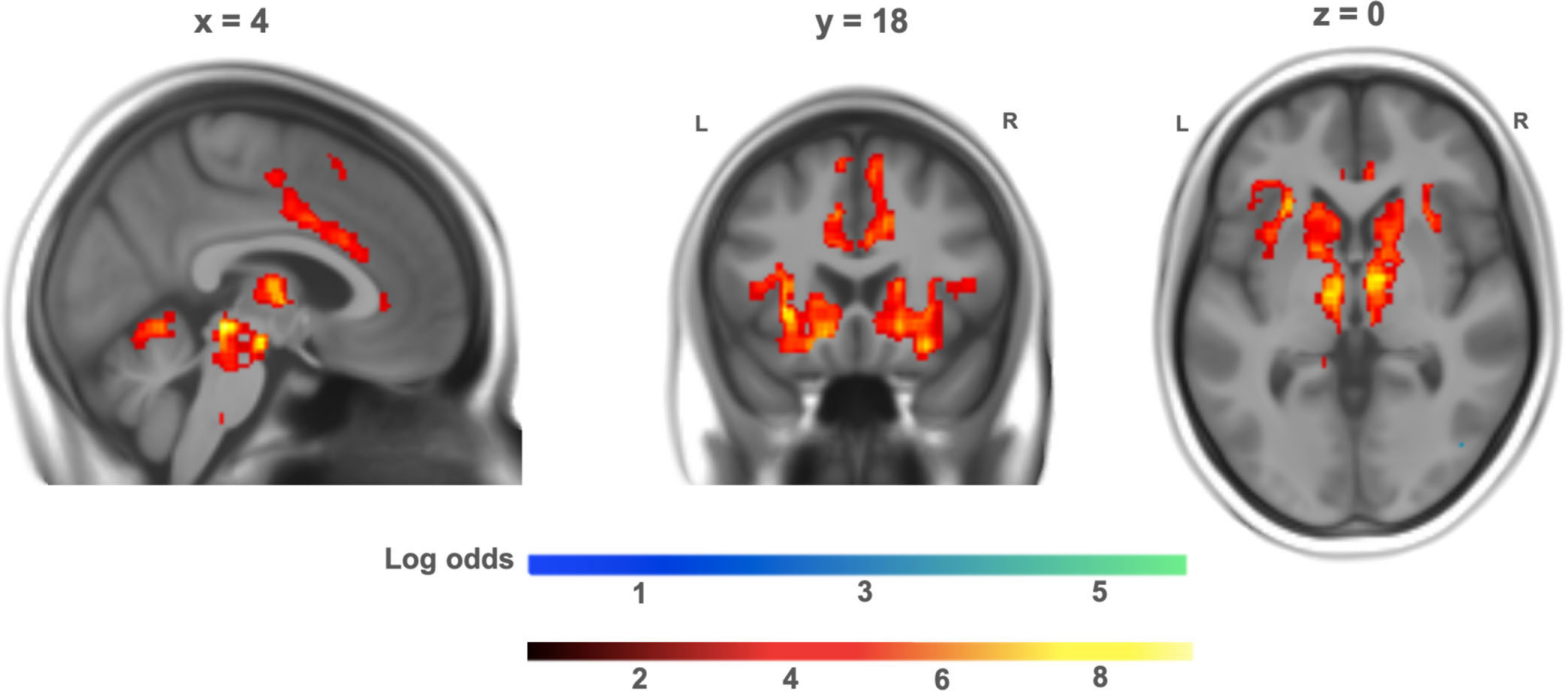
Neural correlates of pupil change within conditions. Hot colors: blood oxygen level dependent (BOLD) activity positively correlated with pupil change. Cold colors: BOLD activity negatively correlated with pupil change (*d* = 0.5, logBF >3). L = left, R = right

The GLM with the mean pupil change values of the one‐second time bins revealed very strong evidence for correlation with BOLD activity in the bilateral insula, caudate, thalamus, orbital inferior frontal gyrus, anterior and middle cingulate gyrus, as well as in the superior frontal gyrus, *d* = 0.5, logBF >3 (Figure 5). The anterior insula and the anterior cingulate gyrus are typically conceptualized as the primary components of the salience network (Menon & Uddin, 2010). The negative contrast revealed strong evidence for corresponding negative correlations with the occipital lobe, *d* = 0.5, logBF >3. For a detailed listing of these clusters, see Table S5 in the Supplement.

To examine the possibility that these correlates (Figure 5) were confounded by differences in the mean pupil change values per condition (main effect of condition on pupil change, posterior probability = 1.0), we ran two additional fMRI analyses. For the first analysis, we entered the *mean* pupil change values of each block (one value per block) into the GLM, analog to the aforementioned pupil size GLM. For the second analysis, we entered demeaned pupil change values within 1 s time bins (40 s per block = 40 values) as parametric modulation in four separate regressors (one per condition) into the model, such that there were no mean differences among the blocks anymore. For the demeaned pupil change values, the mean pupil change of each block was subtracted from the pupil change value in the corresponding 1 s time bins.

If the first analysis would show the salience network but not the second one, this would be evidence for confounding mean differences per block. If the second analysis would show the salience network but not the first, it would be evidence that this network correlates with dynamic within‐block fluctuations. We could observe the latter pattern of results (see Figures S10 and S11 in the Supplement), as the second analyses revealed activation in the bilateral insula, caudate, thalamus, orbital inferior frontal gyrus, anterior and middle cingulate cortex (*d* = 0.5, logBF >3). These clusters (Table S6 in the Supplement) showed a strong overlap with those of our main analysis of pupil change within conditions (Figure 4). This supports the notion that it is not the mean pupil change between conditions, but rather pupil change within (i.e., during) the conditions that drives this result.

To further examine if there was an effect of condition on the strength of the salience network adherence to the pupil change dynamics, we performed an ANOVA testing for the main effect of condition and a conjunction analysis with the three N‐back conditions, as well as all four conditions. In the ANOVA (main effect), we did not observe relevant activity at the set thresholds (voxelwise *p*_FWE_ < .001). To evaluate whether we missed potentially relevant clusters due to an overly conservative threshold for a comparison among conditions (as opposed to a test against 0 as in the previous tests), we reduced the threshold to a frequentist voxelwise *p*_FWE_ < .05, *k* > 50. At this lower threshold, we could observe eight clusters as listed in Table S7. The activation was mainly evident in the bilateral caudate, middle and posterior cingulate gyrus, inferior frontal gyrus, in the middle frontal gyrus (*p*_FWE_ < .05) (Figure S12 in the Supplement). The conjunction analysis encompassing the parametric modulation of mean pupil change of three conditions requiring a response (0‐back, 1‐back, and 2‐back) showed activation in the bilateral insula, SMA and the inferior frontal gyrus and middle cingulate gyrus in the right hemisphere at this lower threshold (voxelwise *p*_FWE_ < .05, k > 30) (Figure S13 in the Supplement). A conjunction analysis of all four conditions (including fixation) only revealed similar activity at a low, uncorrected threshold (uncorrected *p* < .001) (Figure S14 in the Supplement).

**FIGURE 6:**
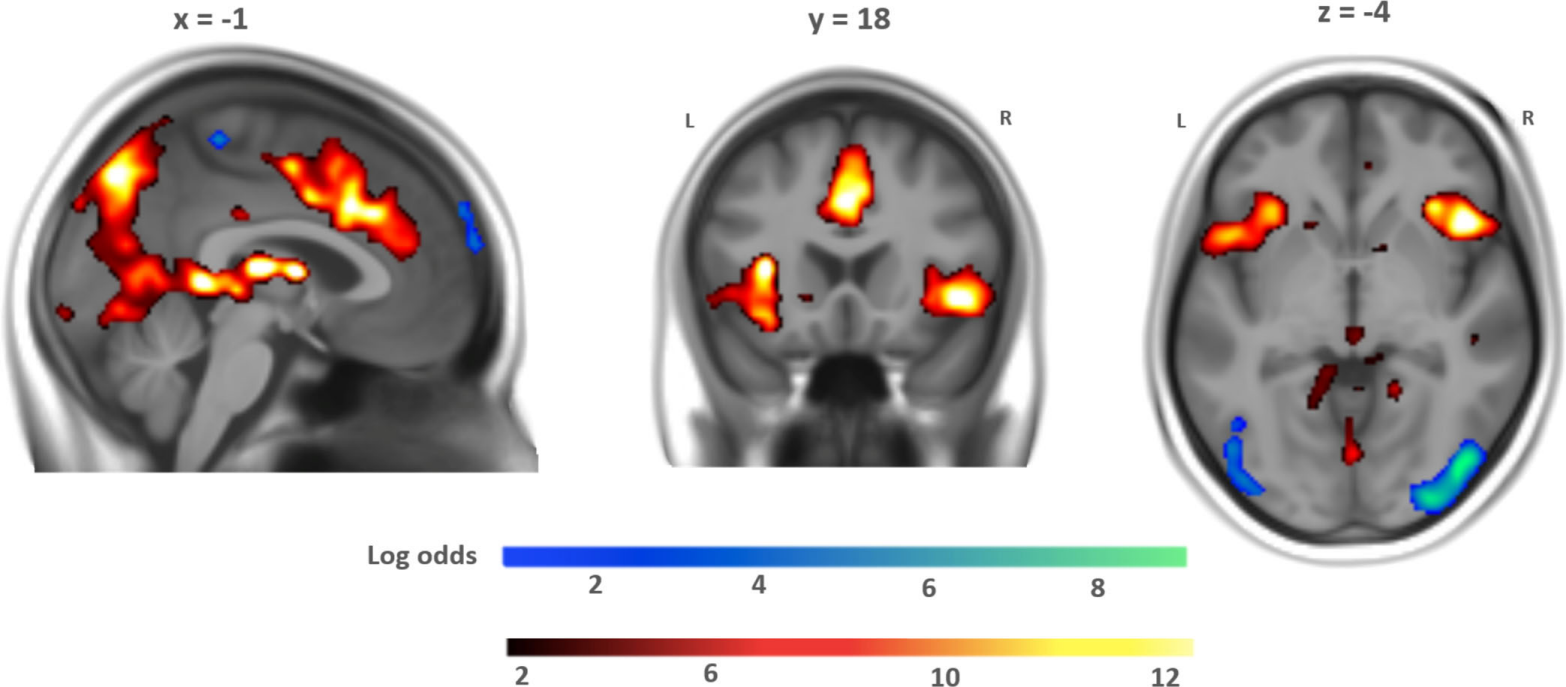
Neural correlates of pupil peak (maximum pupil size value in search window) per trial. Hot colors: blood oxygen level dependent (BOLD) activity positively correlated with pupil peaks per trial. Cold colors: BOLD activity negatively correlated with pupil peaks per trial (d = 0.2, logBF >3). L = left, R = right

The second level GLM of the pupil peak values per trial revealed very strong evidence for positively correlated BOLD activity mainly in the salience network (bilateral insula, dACC), thalamus, and SMA, *d* = 0.2, logBF >3 (Figure 6). The negative contrast revealed strong evidence for corresponding negative correlations mainly within the occipital lobe, *d* = 0.2, logBF >3. For a detailed listing of these clusters, see Table S8 in the Supplement.

#### 3.3.3 Conjunction: Neural correlates of pupil size and pupil change

**FIGURE 7:**
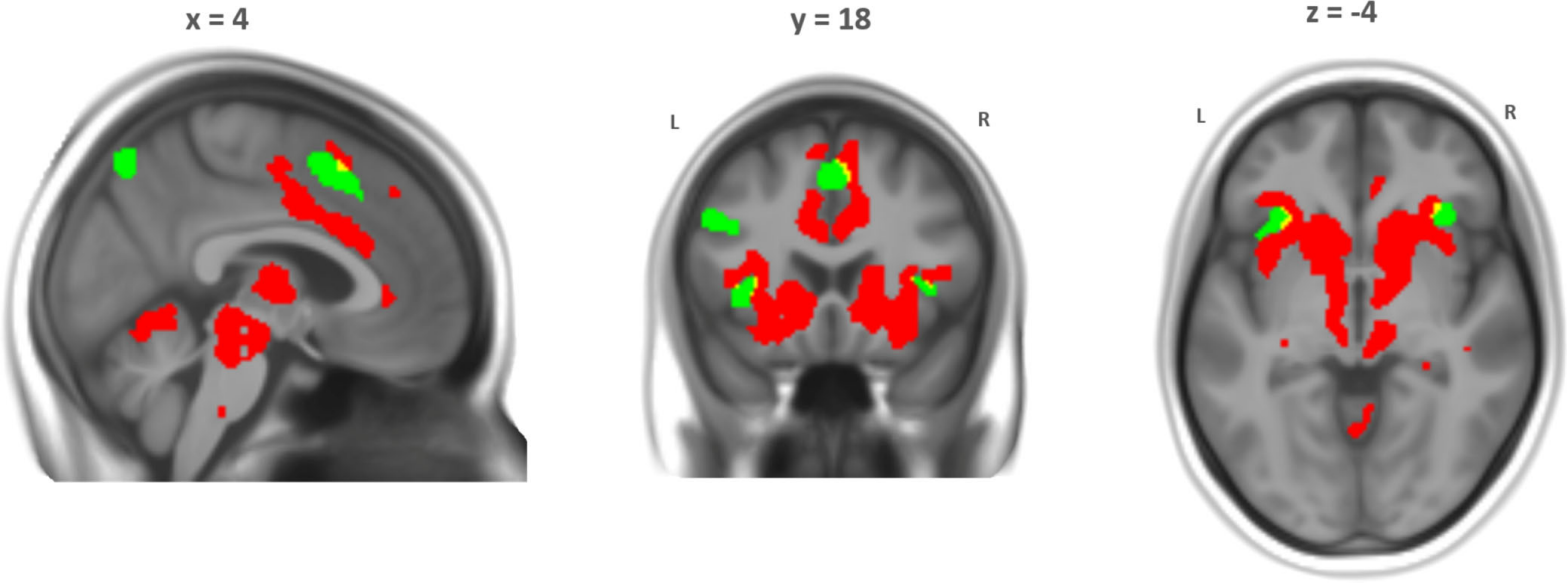
Regional overlap (in yellow) of neural correlates of pupil size *between* condition (in green; *d* = 0.2, logBF >3) and pupil change *within* condition (in red; *d* = 0.5, logBF >3)

We overlaid the contrasts from the analysis on pupil size between conditions and of pupil change within conditions to examine the regional overlap (Figure 7). This conjunction analysis revealed activity in the dACC and bilateral insula for both contrasts. Interestingly, this activity was almost completely nonoverlapping, but rather in adjacent subregions. The between condition pupil size‐FPN network revealed slightly more dorsally located clusters in the dACC/SMA, for instance, whereas the within condition pupil change‐salience network revealed a more ventrally located cluster just above the corpus callosum with associated activity in the midbrain/brainstem, thalamus, and basal ganglia.

However, this apparent separation disappears at more lenient, frequentist thresholds (*p*_FWE.voxel_ < .05 and *k* > 100), with clusters of activity in dACC and bilateral anterior insula becoming overlapping (Figure S15 in the Supplement).

## 4 DISCUSSION

In this study, we investigated the relationship between pupil fluctuations and associated BOLD correlates during working memory processing in healthy humans. For this purpose, participants performed an N‐back fMRI task while their pupil size was recorded simultaneously at a high sampling rate. To couple pupillometry with our fMRI analysis, we quantified pupil fluctuations in two ways: (a) as differences of mean pupil size *between* the N‐back conditions that were characterized by systematically varying working memory load levels, and (b) as pupil change *within* these conditions. Eventually, these extracted pupil size and pupil change measures were entered into separate first level GLMs of the fMRI BOLD time series.

As hypothesized, our results provided strong evidence for an increase in pupil size with increasing working memory load, confirming a robust interrelation between pupil size and the cognitive effort that was encoded in the experimentally controlled levels of working memory load. This aligns well with previous pupillometry reports (Robison & Unsworth, 2019; Unsworth & Robison, 2018) and our behavioral measures that reflected increasing task difficulty with increasing working memory load, as indicated by typical stepwise RT and accuracy profiles. These behavioral results represent an important validation that at the group level, varying difficulty levels could be successfully induced.

Analysis of BOLD activity linked to pupil size differences *between* conditions yielded very strong evidence for activation of the bilateral FPN including the dorsolateral PFC, ventrolateral PFC, posterior parietal lobule, cerebellum and bilateral insula, albeit a small effect size. Considering previous studies of neural activation during working memory processing (Mencarelli et al., 2019; Owen et al., 2005; Rottschy et al., 2012), our findings related to pupil size *between* conditions were in line with the working memory network gained meta‐analytically from 189 studies that revealed a strong, consistent bilateral activation of the FPN encompassing the inferior frontal gyrus, bilateral insula, SMA, superior frontal gyrus, and superior parietal lobule (Rottschy et al., 2012). In the inverse contrast, we observed the default mode network (DMN), the typical task negative network (Raichle, 2015). The results so far support the understanding that pupil size averaged per condition is robustly reflecting the current working memory load at the subject level, similar to analyses that directly model the gradual working memory recruitment of the FPN and DMN (Di, Zhang, & Biswal, 2020).

The question that guided our further analyses, however, was how this can be integrated with the literature on correlations between pupil dilation and the salience network (Leuchs et al., 2017; Schneider et al., 2016, 2018). We addressed this topic by focusing on the neural correlates of mean pupil change *within* conditions and observed very strong evidence with a medium effect size for a positive correlation of pupil change with the activity level of the salience network. We should add that this network, beyond its typical insular and dACC hubs, also relays on the arousal system, such as the thalamus and the posterior cingulate (Menon & Uddin, 2010). This correlation between pupil change (first‐order derivative of pupil size) and the salience network was largely independent of the working memory load level, as our secondary analyses revealed practically equivalent maps of the salience network in 0‐back, 1‐back, and 2‐back conditions (Figure S16 in the Supplement) with minor differential effects between these conditions. When examining the peak voxel contrast estimates the activation in the bilateral caudate, for example, was mostly driven by the 2‐back condition, possibly pointing toward a certain threshold of task complexity that triggers involvement of the caudate only at the most difficult stage (Figure S17 in the Supplement). Thus, we suggest that cognitive load, or simple motor responding, does affect the correlations between pupil change in more peripheral regions of the salience network but not its core regions.

However, it is important to point out that these two distinct subprocesses of working memory also showed a regional overlap and share parts of the activation patterns mainly in the bilateral insula, dACC, even though the peaks were adjacent and largely nonoverlapping within these regions. This conjunction of FPN and salience regions supports the notion that salience network regions, anterior insula and dACC, are involved in both processes: salience detection and cognitive demand. The salience network, and particularly the insula, integrates cognitive information and acts as a switch between large‐scale networks to facilitate access to attention and working memory (Menon & Uddin, 2010). Furthermore, the anterior insula and the dACC exhibit a close functional relationship and are fundamental for effort related processes (Medford & Critchley, 2010). In a wide range of cognitive tasks, including the N‐back task, a coactivation of the salience network and the PFC is very common (Kurth, Zilles, Fox, Laird, & Eickhoff, 2010; Menon, 2011). Our results, based on the simultaneous measurement of pupillometry and fMRI, point toward a physiological upregulation when a target stimulus is detected in a high demand condition and a response is required, through connecting the insula and dACC with arousal‐related regions (brainstem/midbrain, thalamus, and basal ganglia). According to the adaptive gain theory, the LC receives top‐down task‐related information from high‐level structures and anatomical studies have shown cortical projections from the dACC to the LC in primates (Aston‐Jones & Cohen, 2005). The coactivation pattern in our results can be interpreted as a sustained restimulation of the FPN by the salience network, as it is able to lead resources to the FPN. At the same time, the FPN holds the task‐relevant information leading to a potentially stronger interconnection between these two networks.

The fixation condition showed a nonintuitive correlation pattern at first glance: here pupil change revealed a correlation with DMN midline hubs and some overlap with the bilateral dorsal ACC (Figure S16 in the Supplement). The salience network activity was less pronounced compared to positive correlations of pupil change with solely salience network areas during the resting state (i.e., unconstrained cognition) (Schneider et al., 2016), and compared to the 0‐back, 1‐back, and 2‐back conditions in our study. One explanation for the salience network being only weakly coupled with pupil change in the fixation condition might be the lack of salient stimuli and/or goal‐directed motor responses during that block. In turn, one reason for the appearance of the DMN may be the low cognitive demand of the fixation condition (passive viewing of the same repeating stimuli), which is in contrast to the cognitively more demanding N‐back conditions that decrease the DMN “tonically” with less volatility and responsivity to single stimuli. A strong recruitment of the salience network in parallel with pupil dilation seems to occur either at rest (Schneider et al., 2016) when large, low frequency fluctuations are present spontaneously, or in a cognitive context above a cognitive demand threshold that requires actual redistribution of resources from the DMN to FPN. The observed common DMN and salience network recruitment resembles other examples of transient positive coupling between the DMN and other high control networks. Piccoli et al. (2015) reported that during specific subphases of a working memory task—encoding and retrieval—the DMN and the FPN coupled positively, whereas during the maintenance phase with no visual input these networks remained anticorrelated (Piccoli et al., 2015). The salience network plays an important role in promoting such switches (Menon, 2011, 2015; Menon & Uddin, 2010), and our *within* condition results demonstrate that salient stimuli trigger its activity to uphold the functional segregation between the DMN and the antagonistic FPN.

Further, we interpret the differences *between* and *within* condition correlations with pupil size and pupil change as reflecting differences between tonic versus phasic arousal, respectively: The correlation of mean pupil size and activity in the FPN could relate to a tonic pupillary response that increases as the task becomes more challenging. In addition, and occurring concurrently, the active N‐back conditions contain target stimuli conceivably triggering a phasic response that correlates with the salience network independent of working memory load.

Both the tonic and phasic pupillary arousal states could be attributed to the LC norepinephrine (NE) system (Aston‐Jones & Cohen, 2005; Gilzenrat, Nieuwenhuis, Jepma, & Cohen, 2010). The LC is a neuromodulatory nucleus in the brainstem that is responsible for most the NE released in the brain, and it has widespread projections throughout the neocortex including frontal–parietal areas. Pupil dilations related to cognitive processing are thought to result from an inhibitory effect on the parasympathetic oculomotor complex by release of NE from the LC (Szabadi, 2013). A possible explanation for our observation might be that the task demand results in an increased firing rate of LC neurons, which leads to an enlargement of the pupil diameter and facilitation of working memory processing in the PFC areas, which again is interconnected with the LC constituting a reciprocal relationship (Alnaes et al., 2014; Arnsten, Wang, & Paspalas, 2012; Mather et al., 2020; Sara & Bouret, 2012). To date, there are no existing studies explicitly relating LC neuronal activity to working memory, but neuropharmacological studies provide evidence of the essential role of NE release for executive functioning (Arnsten et al., 2012; Ramos & Arnsten, 2007). Hitherto, the increase in pupil size during working memory was associated with task‐evoked phasic arousal, arguing that attention was constantly allocated in order to actively maintain items in working memory (Unsworth & Robison, 2018). We speculate that the LC tonic activity might be responsible for the general increase in the overall pupil size *between* conditions and the LC phasic activity may be related to the pupil change *within* conditions generated by the target stimuli. In the event‐related analyses (Figures 3 and 6), we showed that the within condition pupil responses were specifically related to the trials, which are primarily affected by stimulus type (target vs. non‐target) as larger pupil dilations were associated with target trials and elicited activation in the typical salience brain regions. Interestingly, the pupil dilation in response to target trials was larger for the trials with lower cognitive load. This is most likely a consequence of the larger mean pupil size in the higher cognitive load conditions (Peysakhovich, Vachon, & Dehais, 2017).

This raises the question of whether these stimulus type driven modulations are associated with stimulus saliency or effort allocation. In our N‐back task, the target and non‐target trials were not distinguishable by visual features alone. The participants needed to constantly update their information in mind and then identify the target solely by correctly memorizing the preceding trials (and in the case of the 0‐back condition remembering one specific stimulus), meaning that the identification of the salience of targets required task‐engagement and effort allocation toward the stimulus. Research on primates has shown that the phasic response is not particularly linked to specific sensory attributes of a stimulus, but rather to task‐relevant events (Aston‐Jones & Cohen, 2005). Following this line of thought, it is possible that the effort allocation precedes the experienced salience of the target, and the resulting correlation with the salience brain networks is a product of both processes (Engström, Landtblom, & Karlsson, 2013).

The relationship of pupil fluctuations and neural activity is probably not exclusively dependent on the LC and the general noradrenergic tone controlled by it. Electrophysiological research in rhesus monkeys has pointed toward a similar relationship of pupil temporal dynamics and the inferior and superior colliculus in the mesencephalon. Additionally, neural activity in the dACC could also be aligned in time with changes in pupil diameter, reflecting underlying changes in arousal (Joshi, Li, Kalwani, & Gold, 2016).

Murphy et al. (2014) observed a very similar pattern of activation as they also found positive correlations of pupil diameter with activity in ACC, insula, and the thalamus in an oddball task. Moreover, they could show that pupil diameter was positively correlated with BOLD activity in the rostral LC (peri‐LC), providing the first fMRI evidence supporting the notion that the pupil diameter can be used as indirect measure of LC activity (Murphy et al., 2014). In our analyses of pupil change *within* conditions, we also observed activity in the brainstem in areas that could encompass the LC, but our resolution and preprocessing was not optimized for brainstem analyses.

Although the N‐back task is well‐established and has been one of the most commonly used experimental paradigms for exploring the neural basis of working memory and executive functioning (Lamichhane, Westbrook, Cole, & Braver, 2020), there are a few methodological considerations with respect to our interpretations. The blocks of the N‐back task utilized in this study did not follow a randomized order, which means that theoretically the fixed order could have an influence on the results. Nevertheless, as each condition was present once in the first half and once in the second half of the task, and as no tiring effect was detected in the pupil data, we assume that the influence of the design limitation was marginal (for analysis, see section 2.3 in the Supplement). Another restraint may lie in the conditions of the task itself, which is noticeable in the accuracy rates that showed overall a very high level of correct responses. Although we observed large differences in accuracy between 1‐back and 2‐back, and 0‐back and 2‐back, we did not observe a difference between the 0‐back and 1‐back condition in accuracy rates. This is probably due to a ceiling effect, with similar patterns observed in healthy subjects in previous work (Hur, Iordan, Dolcos, & Berenbaum, 2017; Jacola et al., 2014). These authors have proposed that RTs represent a more meaningful readout for the N‐back task. In our study, we could observe a difference between all conditions regarding that measure. The condition with the maximum working memory load was 2‐back, and conditions with higher load are generally feasible in healthy subjects. The reason for not including a 3‐back condition is that our task is part of a larger study on psychiatric patients, some of which have mood disorders with cognitive impairments, and healthy participants. However, when taking our pupillary, behavioral and the neural readouts into account, we can safely claim that the working memory manipulation was successful, similar to previous work that did not include a 3‐back condition (Alonso‐Lana et al., 2016; Dores et al., 2017; Peysakhovich et al., 2017). Nevertheless, future research could incorporate conditions with higher load in order to be able to observe a potential inverted U‐shaped pursuant to the Yerkes–Dodson law (Yerkes & Dodson, 1908). Prior research on this has shown, that pupillary dilation during a working memory task increased until it reached an asymptote at around four to five items held in mental storage (Robison & Unsworth, 2019; Unsworth & Robison, 2018). This could be of potential interest as previous research has related this pattern to the influence of NE on PFC functioning. In their work, the NE release was dose dependent and also followed an inverted U relationship, suggesting that performance increases with physiological or mental arousal, but only up to a certain point until it reaches a plateau before starting to decline (Aston‐Jones & Cohen, 2005; Sara & Bouret, 2012).

To summarize, our findings suggest that fMRI with simultaneous measurement of pupil parameters constitutes a valuable experimental setup to decipher cognitive processes related to working memory load itself versus the immediate salience of the presented stimuli. This distinction could be specifically relevant for patients with psychiatric disorders. Cognitive impairment and in particular, working memory deficits manifest in a wide range of psychiatric disorders both of the affective and psychotic spectrum (Snyder, 2013). It has thus been proposed as a transdiagnostic endophenotype or risk factor (Nolen‐Hoeksema & Watkins, 2011). Similarly, the salience network has been identified as critical to psychiatric disease susceptibility (Goodkind et al., 2015) across the affective and psychosis spectrum, and as such, combined, sensitive tools for studying working memory processes and their link to salience activity are particularly relevant. To this notion we add, that two working memory subprocesses related to cognitive load and salience could be distinguished by parallel fMRI and pupillometry, which could help develop a more valid biological characterization of working memory processes and deficits.

## 5 CONCLUSION

Incorporating pupillometry in fMRI measurements during a working memory task allowed differentiation between working memory load effects and effects of the salience of the presented stimuli. We demonstrated, that the mean pupil size *between* condition was related to the FPN and that pupil change *within* conditions was associated with activity in the salience network, independently from working memory load. This combination of pupil and fMRI parameters may constitute an effective tool for disentangling working memory subprocesses that could be relevant for a range of psychopathological conditions.

## CONFLICT OF INTEREST

The authors declare no potential conflict of interest.

## AUTHOR CONTRIBUTIONS

**Julia Fietz**: Methodology, software, formal analysis, data curation, writing ‐ original draft, writing—review and editing, visualization. **Dorothee Pöhlchen**: Software, formal analysis, writing—review and editing. **Florian P. Binder**: Software, writing—review and editing. **BeCOME Working Group**: Investigation, resources, project administration. **Michael Czisch**: Conceptualization, methodology, resources, writing—review and editing. **Philipp G. Sämann**: Conceptualization, methodology, software, resources, writing—original draft, writing—review and editing. **Victor I. Spoormaker**: Conceptualization, methodology, resources, writing—original draft, writing—review and editing, supervision.

## Supporting information


**Appendix** **S1**: Supporting information

## Data Availability

The data were collected as part of the ongoing BeCOME project (Brückl et al., 2020) at the Max Planck Institute of Psychiatry in Munich, Germany. The data that support the findings of this study are available from the corresponding author upon reasonable request. The unthresholded statistical maps from the second level analyses are available on NeuroVault (https://identifiers.org/neurovault.collection:11191).
