# Supplementary material for: Pupillometry tracks cognitive load and salience network activity in a working memory functional magnetic resonance imaging task
Source: Hum Brain Mapp. 2021 Oct 8;43(2):665–80. doi: 10.1002/hbm.25678 (PMC8720183; doi:10.1002/hbm.25678)
Supplement: Supplementary file 1 — Appendix S1: Supporting information [file HBM-43-665-s001.docx]

SUPPORTING INFORMATION

Pupillometry tracks cognitive load and salience network activity in a working memory fMRI task

Julia Fietz^1,2^, Dorothee Pöhlchen^1,2^, Florian P. Binder^1,2^, BeCOME working group^1,3*^, Michael Czisch^3^, Philipp G. Sämann^3^, Victor I. Spoormaker^1#^

^1^Department of Translational Research in Psychiatry, Max Planck Institute of Psychiatry, Munich, Germany

^2^International Max Planck Research School for Translational Psychiatry (IMPRS-TP), Max Planck Institute of Psychiatry, Munich, Germany

^3^Max Planck Institute of Psychiatry, Munich, Germany

* BeCOME working group:

Elisabeth B. Binder, Tanja M. Brückl, Angelika Erhardt, Norma C. Grandi, Sanja Ilic-Cocic, Susanne Lucae, Alina Tontsch, Julius Ziebula

^#^ Corresponding author: Victor Spoormaker, https://orcid.org/0000-0002-4882-0572 spoormaker@psych.mpg.deDepartment of Translational Research in Psychiatry
Max Planck Institute of Psychiatry
Kraepelinstraße 2-10
80804 Munich, Germany

spoormaker@psych.mpg.de

# SUPPLEMENTARY METHODS

## 1.2 fMRI imaging parameters

The measurements included a specific single spin-echo EPI T2-weighted volume with the same settings as the fMRI sequence, but a longer repetition time (TR = 10 s) and a minimum echo time of TE = 37.1 ms. The resulting single spin-echo EPI volume shows the same geometric distortions as the fMRI time series, due to the identical k-space sampling characteristics, combined with a high signal-to-noise ratio and less signal drop-out. The image was used for segmentation and subsequent normalization to correct for field distortions.

# Supplementary Results

## 2.1 Error-related analyses: behavioral, pupillometry, and fMRI

With regard to the analysis on error responses, we specifically looked at missed hits (= omission errors, not pressing a button in response to targets) and false alarms (= wrongly pressing button in response to non-targets). Both errors were more frequent in the 2-back condition (Figure S1). For missed hits, the rmANOVA revealed a significant effect of condition (*F*_(2,96)_ = 6.01, p = .003) as well as for false alarms (*F*_(2,96)_ = 18.39, p < .001). For the missed hits, post hoc analyses indicated that the average number of missed hits in the 0-back condition (M = 0.63, SD = 1.19) did not differ significantly from the 1-back condition (M = 0.61, SD = 0.84, t = 0.09, p = .92), but the 0-back condition differed significantly from the 2-back condition (M = 1.27, SD = 1.6, t = -2.96, p = .009), and the mean of missed hits in the 1-back condition was significantly different from the mean in the 2-back condition (t = -3.05, p = .009). Post-hoc analyses for false alarms showed as similar pattern since observed a trend for a significant difference between 0-back (M = 0.02, SD = 0.14) and 1-back (M = 0.35, SD = 0.75, t = -1.94, p = .055), but significant differences between 0-back and 2-back (M = 1.02, SD = 1.2, t = -5.95, p < .001) and between 1-back and 2-back (t = -4.0, p < .001).

**
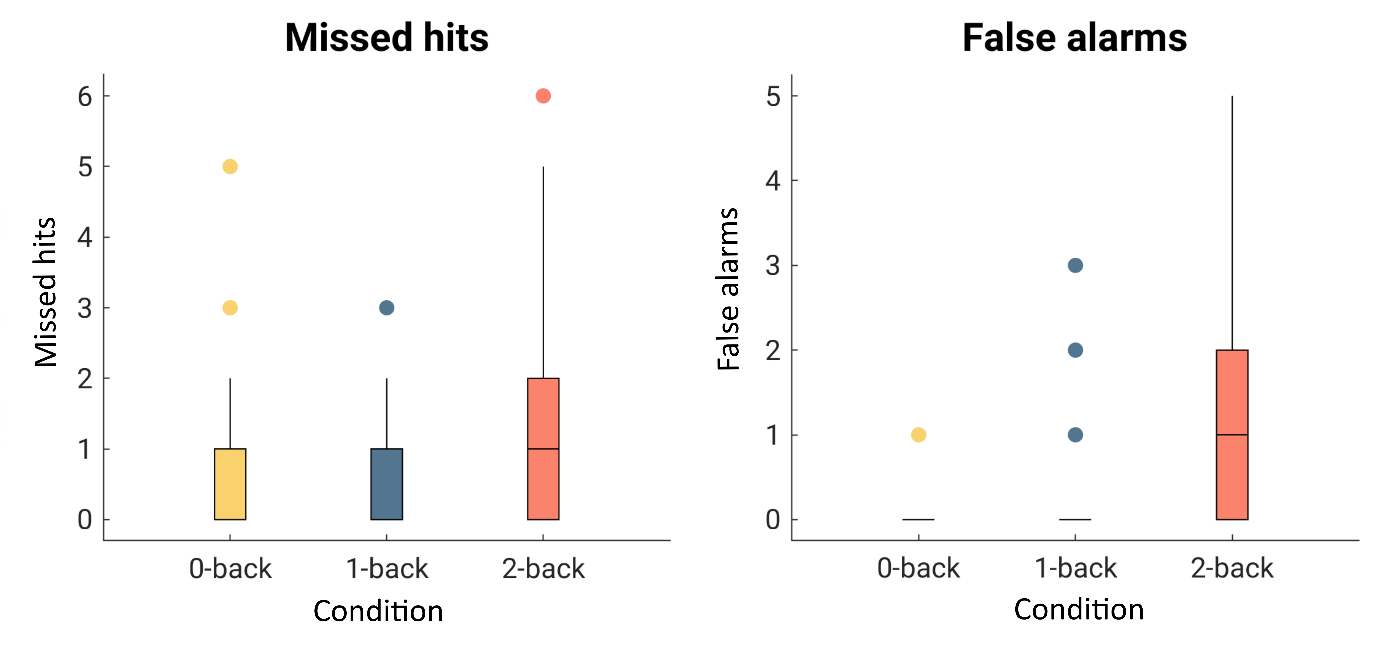
**

**FIGURE S1** Boxplots showing missed hits and false alarms in the three active N-back task conditions (0-back, 1-back, and 2-back)

Regarding missed hits, the analysis on pupillometry indicated a typical error response in the 0-back and 1-back condition, with a trend towards larger pupil dilation in error trials (Figure S2) (Maier et al., 2019; Rondeel et al., 2015). In the 2-back condition, participants potentially paid less attention to target trials as they most likely missed to identify the target and did not respond adequately, which is also indicated by a smaller pupil response and an inversed pattern compared with the 0-back and 1-back condition (Figure S2).

**
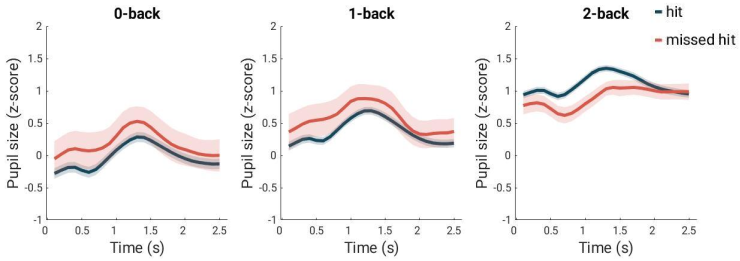
**

**FIGURE S2** Mean pupil size in response to target trials in the three active N-back task conditions (0-back, 1-back, and 2-back). Blue illustrates the mean over all target trials with correct responses (=hits). Red illustrates the mean over all target trials with no responses (=missed hits). The x-axis represents the length (2.5 s) of one trial. Between 0 - 0.5 s the stimulus is presented, between 0.5 - 1.5 s the response if necessary is collected and between 1.5 - 2.5 s is the inter trial interval. The shaded area represents 95% confidence intervals of the mean

For false alarms, we observed a larger pupil size for error trials (response in non-target trials = false alarms) than in trials in which non-targets were correctly identified and no response was given (= correct rejections) independent of working memory load (Figure S3). This is in line with previous literature, showing a stronger pupil dilation in error trials than after correct responses due to error-induced increase in physiological arousal (Compton et al., 2021; Maier et al., 2019; Rondeel et al., 2015).


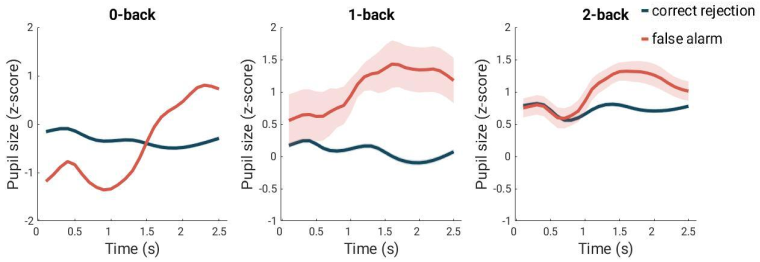
**FIGURE S3** Mean pupil size in response to non-target trials in the three active N-back task conditions (0-back, 1-back, and 2-back). Blue illustrates the mean overall non-target trials with no responses (=correct rejections). Red illustrates the mean overall non-target trials with responses (=false alarms). The x-axis represents the length (2.5 s) of one trial. Between 0 - 0.5 s the stimulus is presented, between 0.5 - 1.5 s the response if necessary is collected and between 1.5 - 2.5 s is the inter trial interval. The shaded area represents 95% confidence intervals of the mean. If no shaded area is present, then it could not be calculated due to insufficient amount of data points

For the fMRI analyses, we created two separate GLMs: one with onsets of all target trials (4 per each block in the conditions 0-back, 1-back, and 2-back) with a duration of 2.5 s. Hits and missed hits were added as a parametric modulation with values of 0 and 1, respectively. The second GLM included onsets of all non-target trials (12 per each block in the conditions 0-back, 1-back, and 2-back) with a duration of 2.5 s. Here, the correct rejections and false alarms were added as a parametric modulation coded in the same manner (0 for correct rejections and 1 for false alarms).

Both missed hits (Figure S4a) and false alarms (Figure S4b) are correlated to activity in the dACC, however the correlations for false alarms reveal the typical salience network with the bilateral insula, whereas salience ‘network’ activation appears somewhat less pronounced in the missed hits contrasts.

**
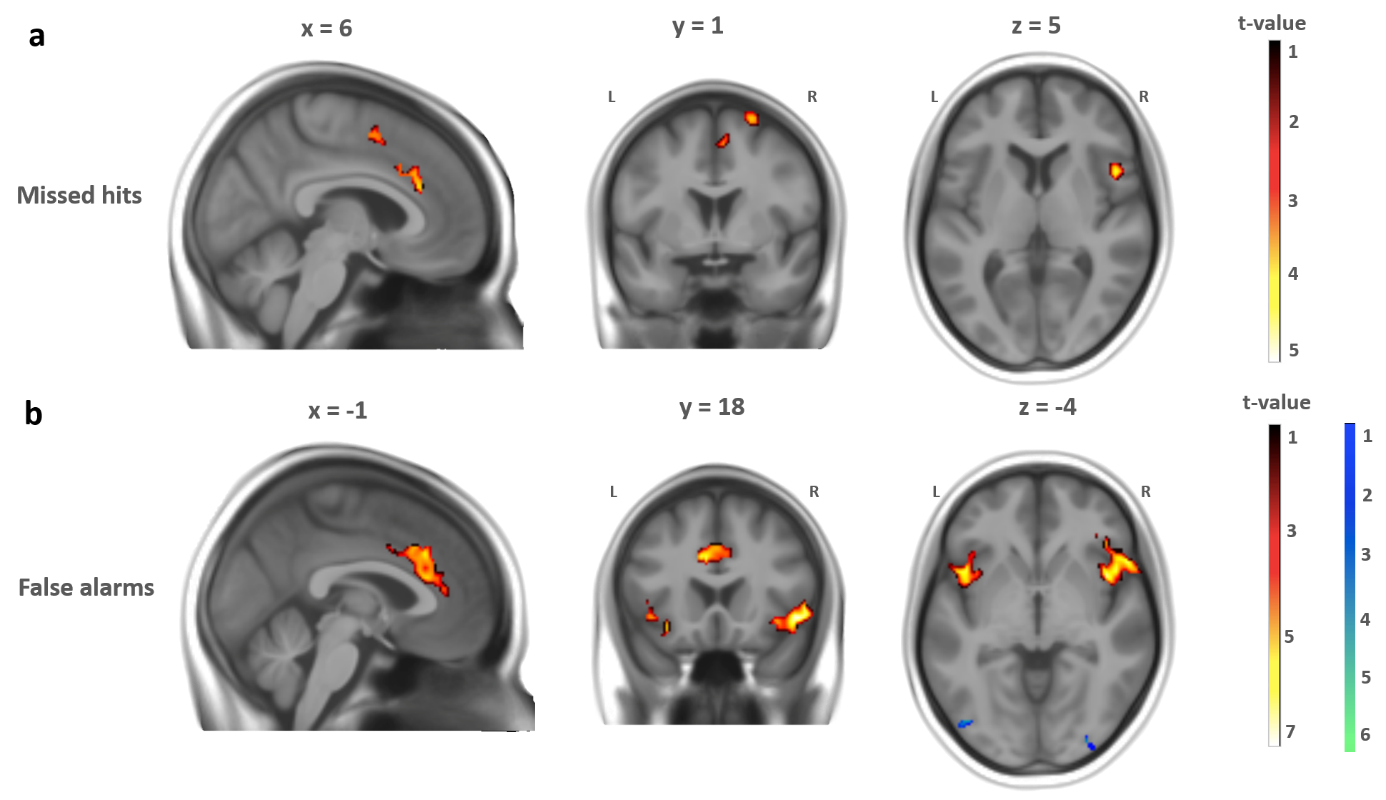
**

**FIGURE S4** a) Neural correlates of missed hits (= omission errors, not pressing a button in response to targets) (uncorrected p < .001, k > 50) and b) false alarms (= wrongly pressing button in response to non-targets) (uncorrected p < .001, k > 50). Hot colors: positive correlation with BOLD activity. Cold colors: negative correlation with BOLD activity. L = Left, R = Right

## 2.2 Results of the main analysis including the initially excluded subjects based on their pupil data

We re-ran the analysis *neural activity related to pupil size between conditions* and *neural activity related to pupil change within conditions* adding the participants that were initially excluded due to our cut-off criteria for the pupil data to check for a potential bias (*n* = 52 + 10). The results showed activation patterns in the same clusters as the main analyses in the manuscript (Figures S5 and S6).


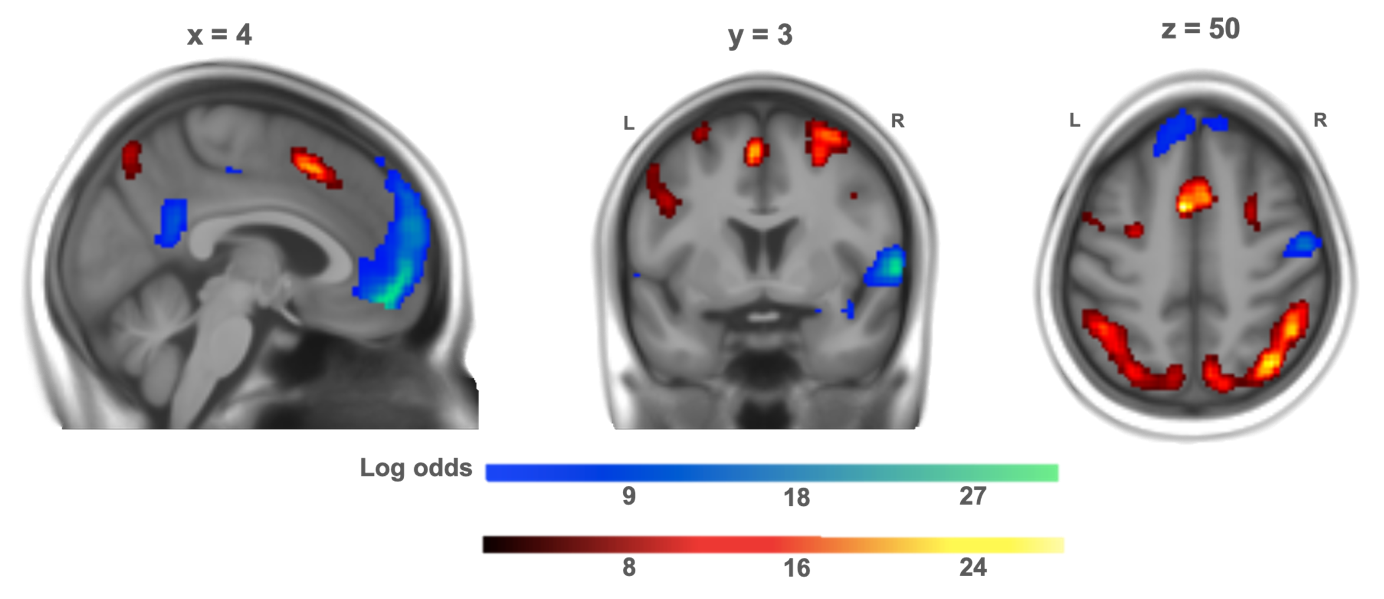
**FIGURE S5** Neural correlates of pupil size between conditions in a larger sample (*n* = 62). Hot colors: BOLD activity positively correlated with pupil size. Cold colors: BOLD activity negatively correlated with pupil size (*d* = 0.2, logBF > 3). L = Left, R = Right

**
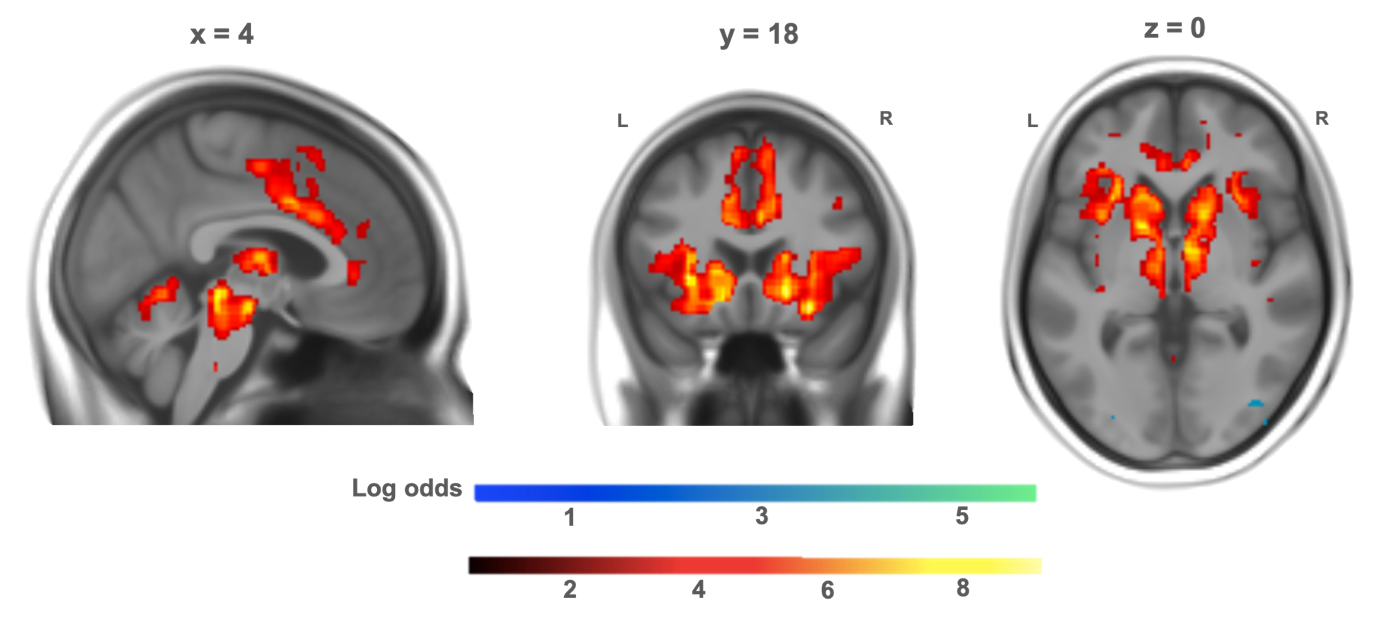
**

**FIGURE S6** Neural correlates of pupil change within conditions in a larger sample (*n* = 62). Hot colors: BOLD activity positively correlated with pupil change. Cold colors: BOLD activity negatively correlated with pupil change (*d* = 0.5, logBF > 3). L = Left, R = Right

## 2.3 Tiring effect between first and second half of the N-back task

We performed an additional analysis in order to check whether study participants became more tired (= tiring effect) between the first and the second half of the task, which would manifest in a smaller pupil size in the second half of the task. For this purpose, we used the mean pupil size of each block and compared the mean values for each condition of the first half with the mean values of the respective condition in the second half of the task using the Bayesian paired samples t-test with a default effect size prior (Cauchy scale 0.707). We found moderate evidence for no difference in the fixation condition (t_(51)_ = -0.75, p = 0.46, BF_10_ = 0.2), very strong evidence for a difference in the 0-back condition (t_(51)_ = -4.37, p < .001, BF_10_ = 359.48, with the second half exhibiting a larger pupil size than the first half), very strong evidence for a difference in the 1-back condition (t_(51)_ = -4.42, p < .001, BF_10_ = 420.5, with the second half exhibiting a larger pupil size than the first half), and moderate evidence for a difference in the 2-back condition (t_(51)_ = -2.85, p = 0.002, BF_10_ = 5.56, the second half exhibiting a larger pupil size than the first half). The results of the descriptive statistics are shown in Table S9.


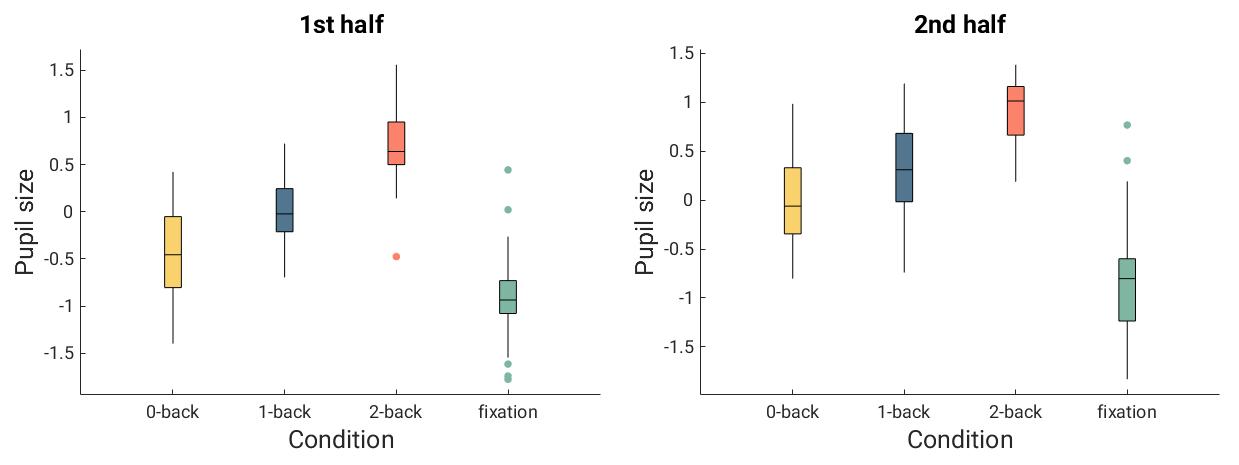


**FIGURE S7** Boxplots showing the distribution of the mean pupil size values (one value per participant) in the first half and the second half of the task

# Supplementary figures


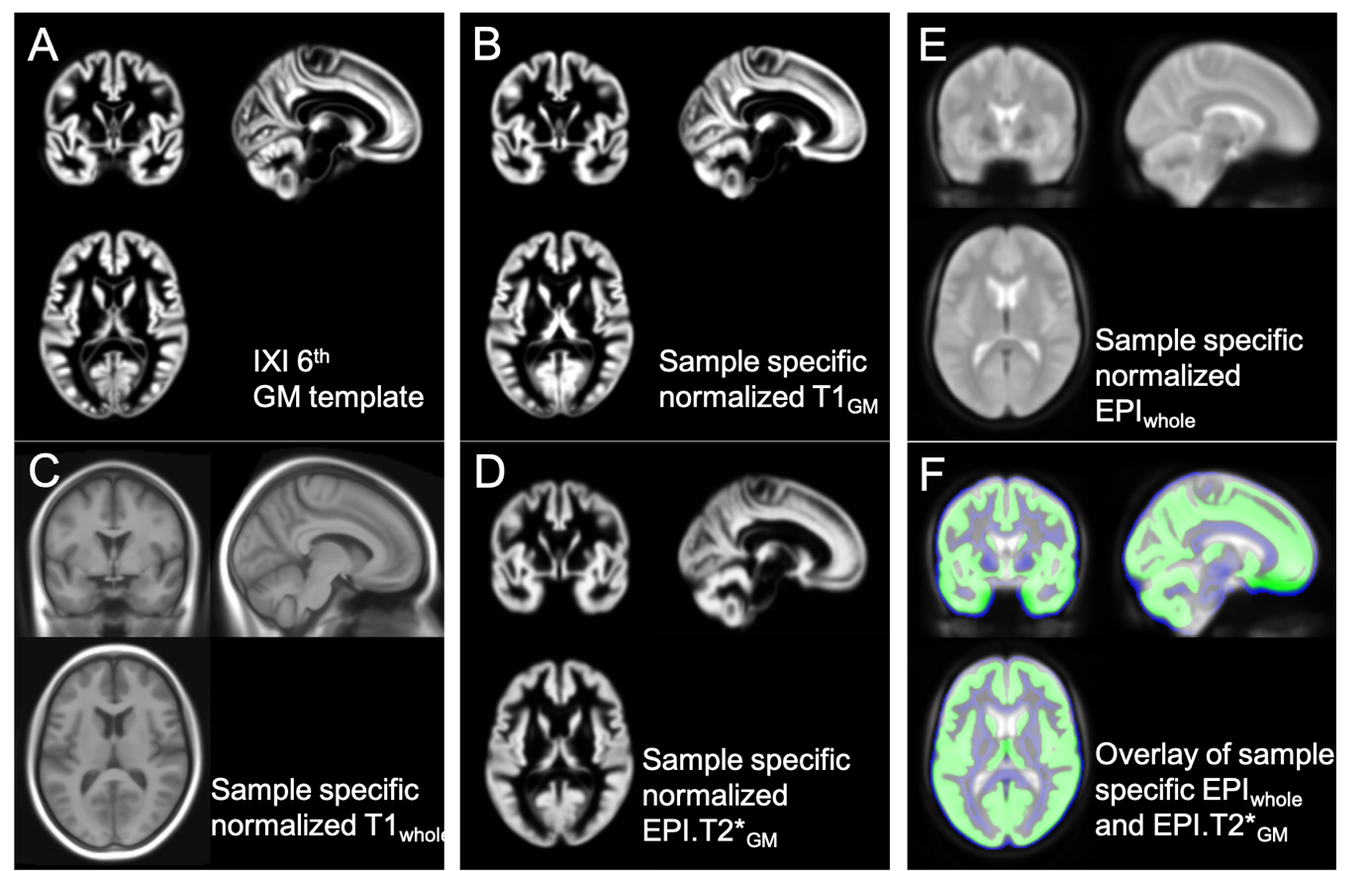


**FIGURE S8** Template and sample specific T1 and EPI normalization results. The following images at the same slice position of [-6 0 10] in MNI space are depicted: (A) IXI GM template (6^th^ generation), (B) sample specific mean of GM segments based on the T1WI, (C) sample specific whole head average used as background image for fMRI results, (D) sample specific GM segments based on EPI.T2* image (E) sample specific mean of whole head EPI images (prior to smoothing, after bias correction), (F) overlay of (E) and (D) with the latter thresholded at 0.5. T1WI = T1-weighted images

**
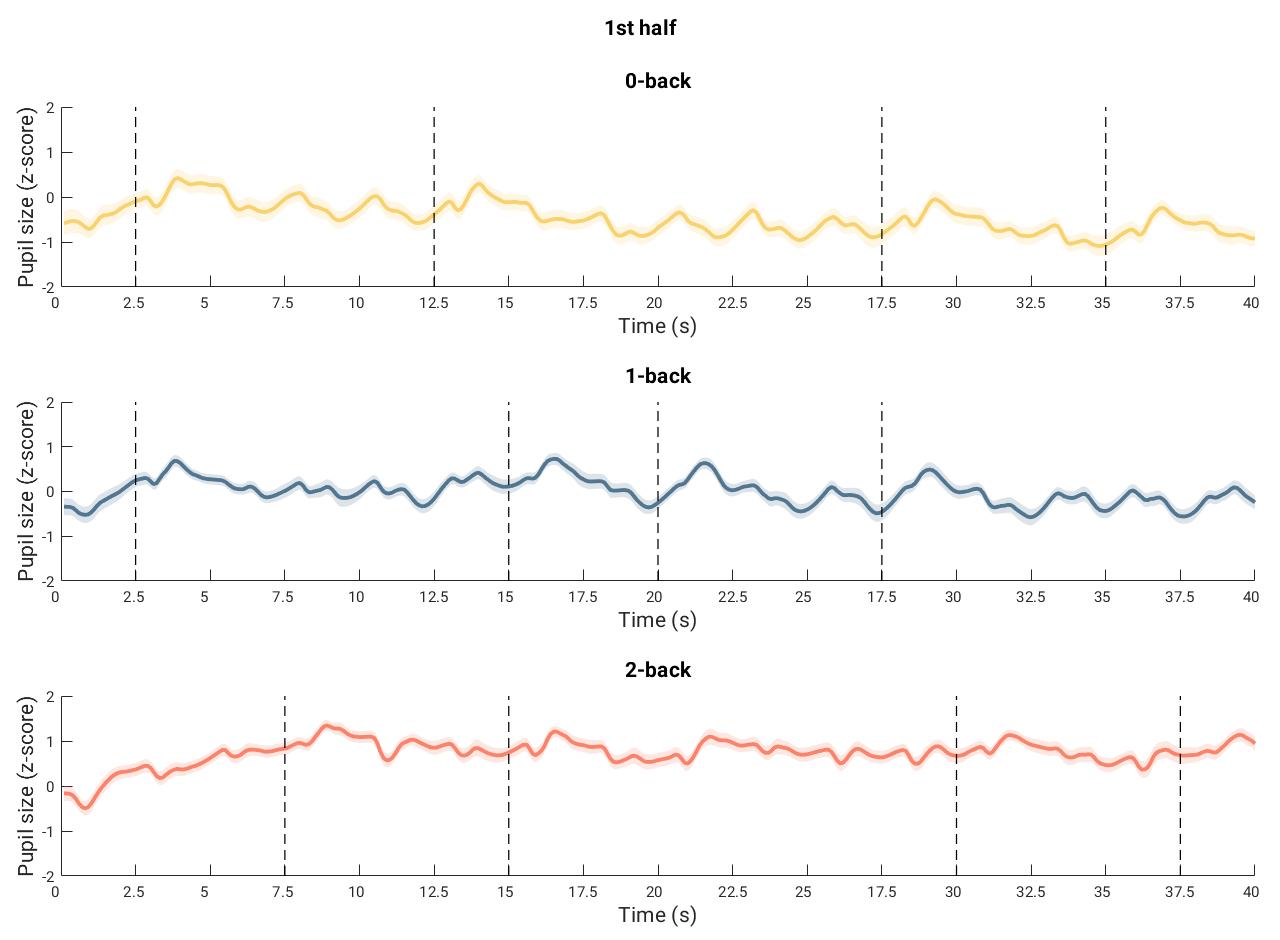
**

**
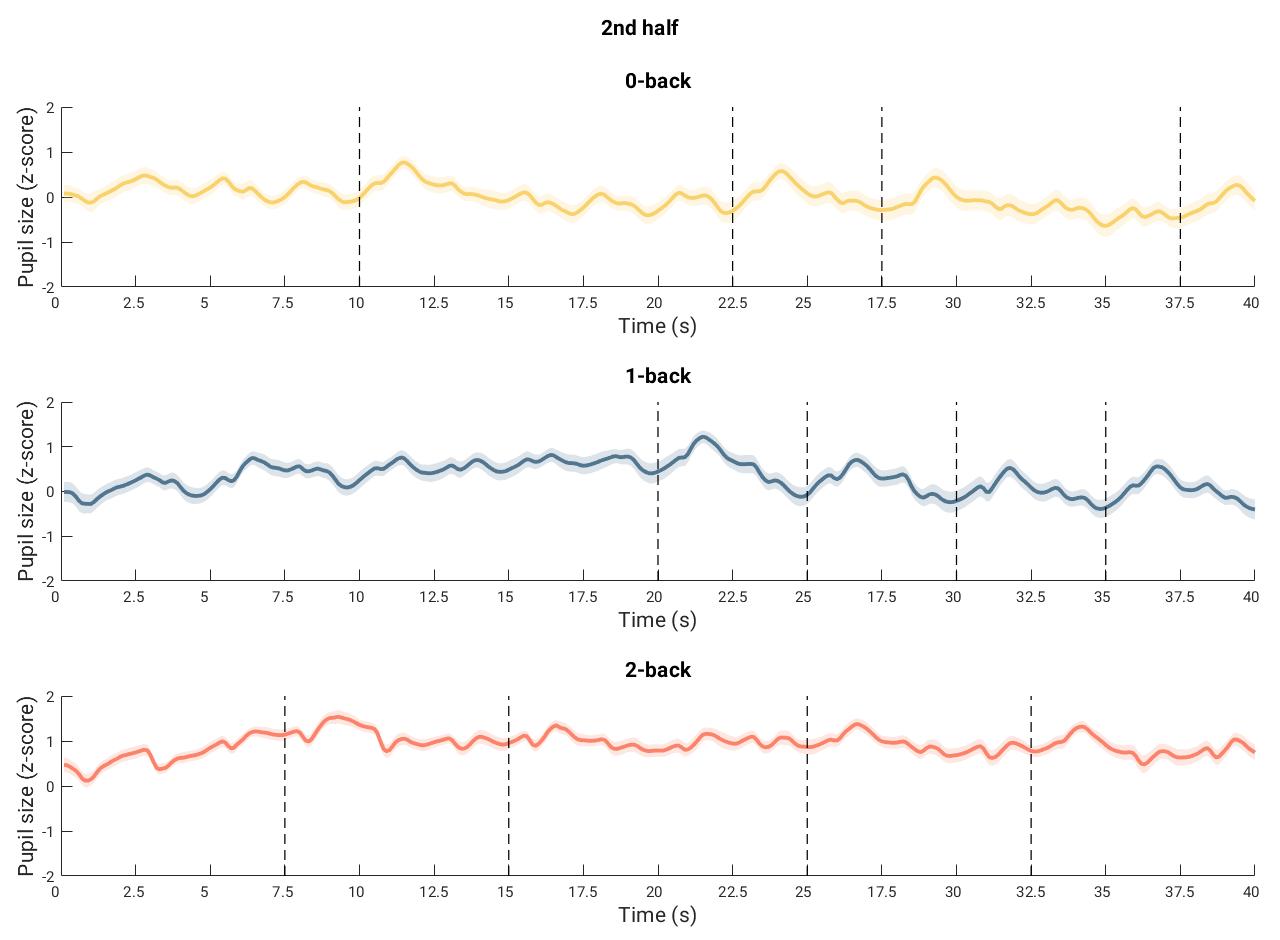
**

**FIGURE S9** Mean pupil size over the time course of a task block within each active task condition divided into first and second half of the task. The x-axis represents the length (40 s) of one block. We down sampled the pupil size values to 10 Hz and calculated the mean of both halves of the task within the conditions. The shaded area represents 95% confidence intervals of the mean. The grey vertical lines indicate target trial onsets, after which larger pupil dilations are detectable

**
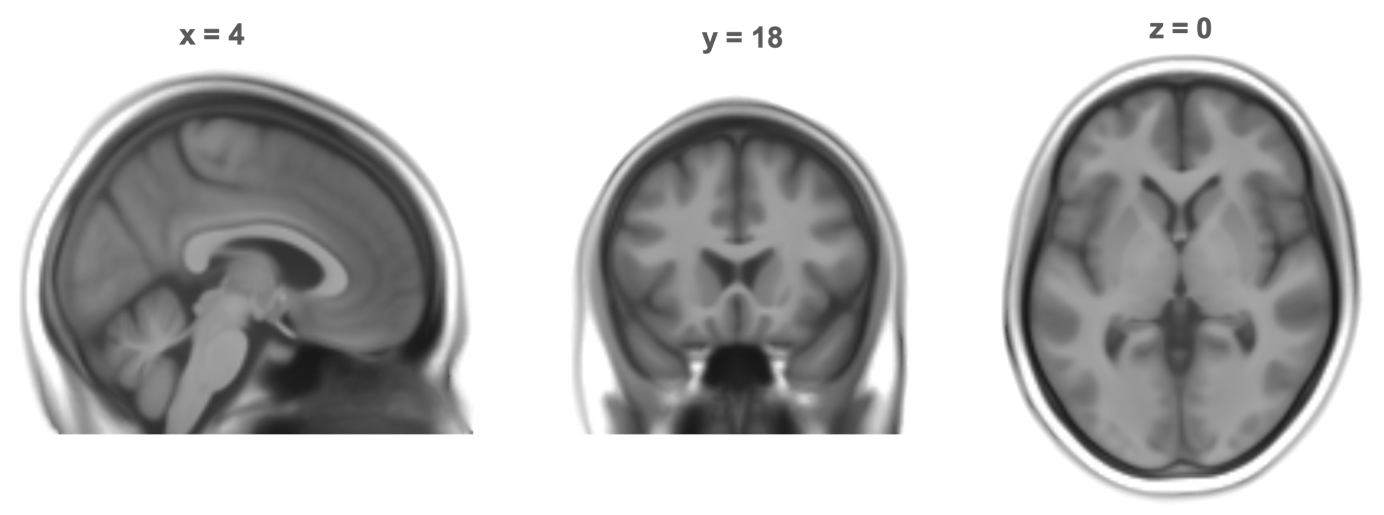
**

**FIGURE S10** Neural correlates of mean pupil change values of 40-second time bins per block / condition. (*d* = 0.2, logBF > 3), L = Left, R = Right


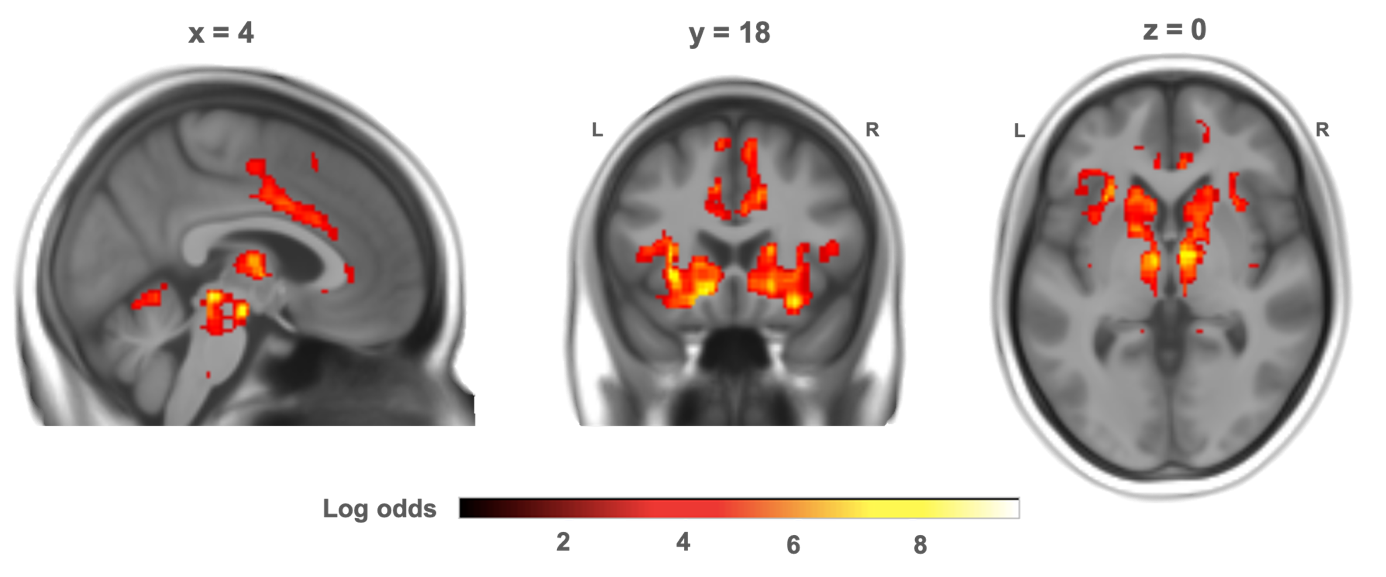
**FIGURE S11** Neural correlates of demeaned pupil change values of one-second time bins. (*d* = 0.5, logBF > 3), L = Left, R = Right

*
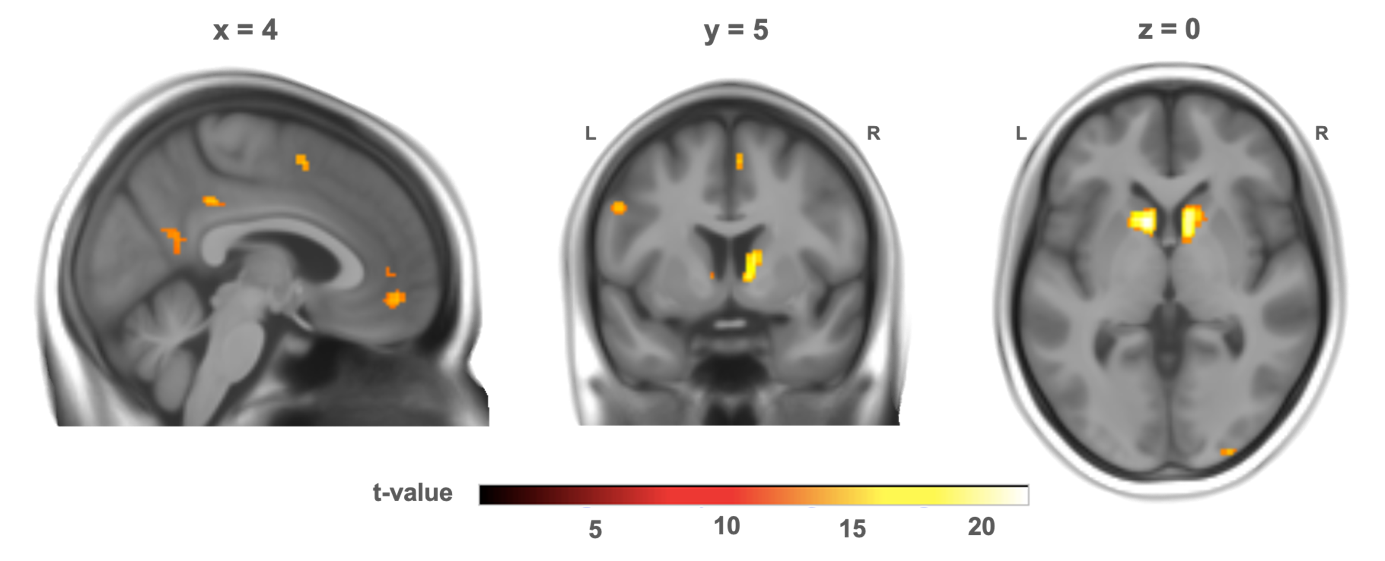
*

**FIGURE S12** Results of the main effect of the ANOVA on pupil change (voxel-wise p_FWE_ < .05). L = Left, R = Right


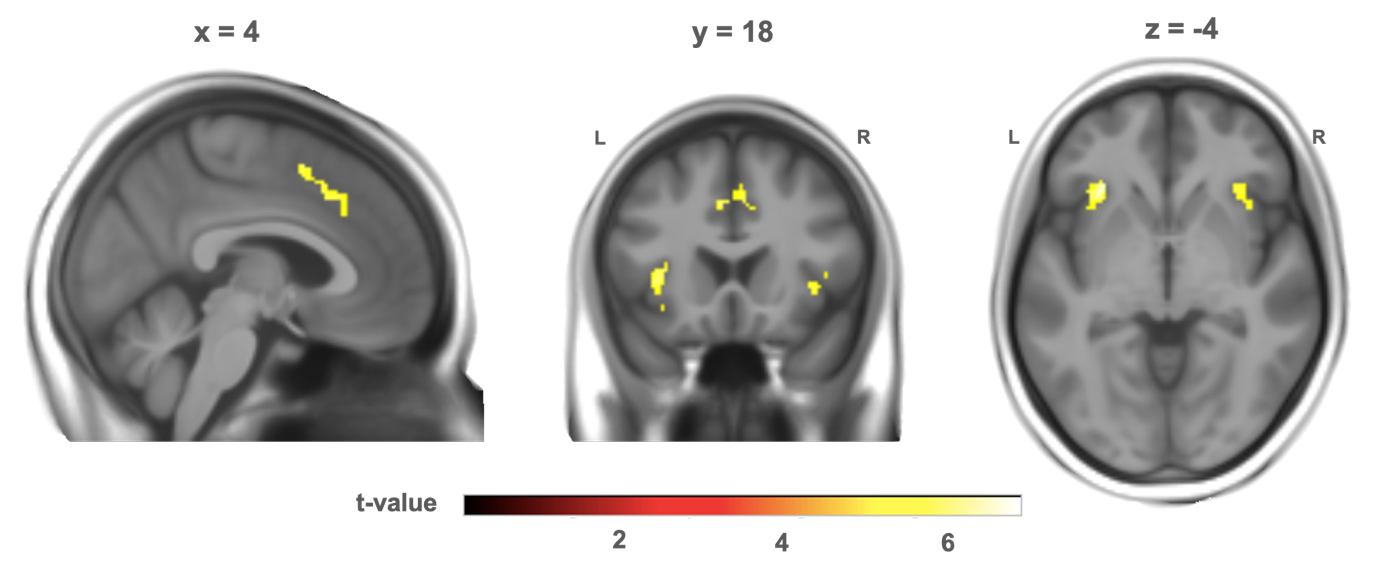


**FIGURE S13** Results of the conjunction analysis on pupil change in three conditions (0-back, 1-back, 2-back) (voxel-wise p_FWE_ < .05). L = Left, R = Right

*
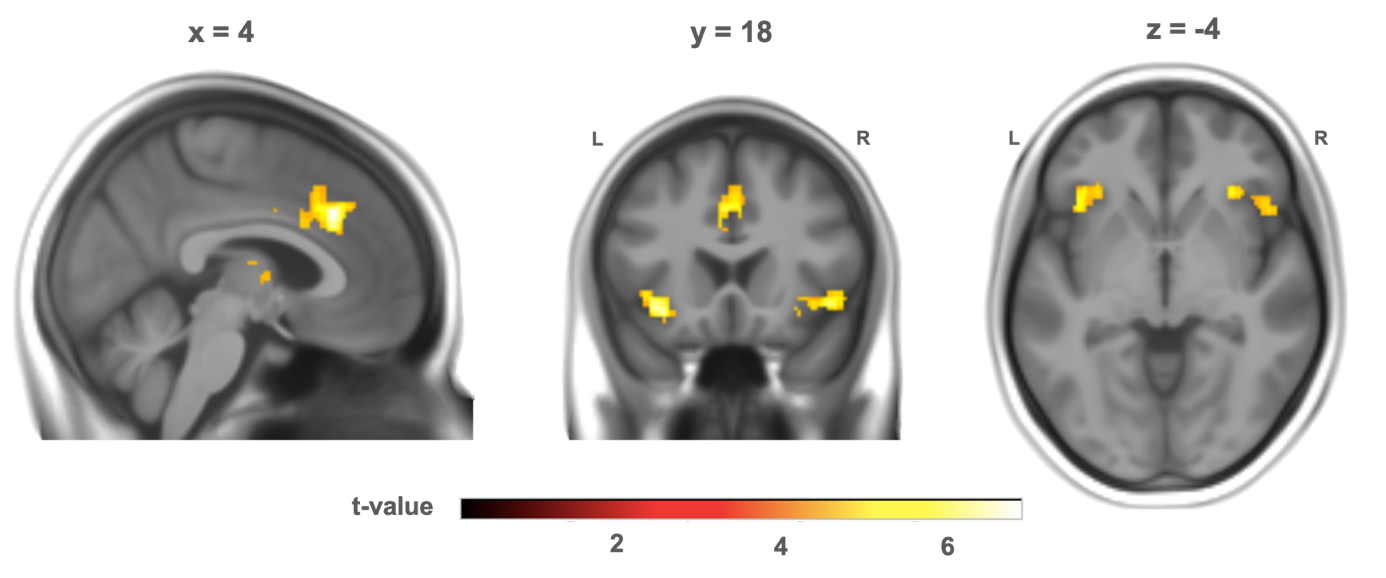
*

**FIGURE S14** Results of the conjunction analysis on pupil change in four conditions (0-back, 1-back, 2-back, fixation) (uncorrected p < .001). L = Left, R = Right

**
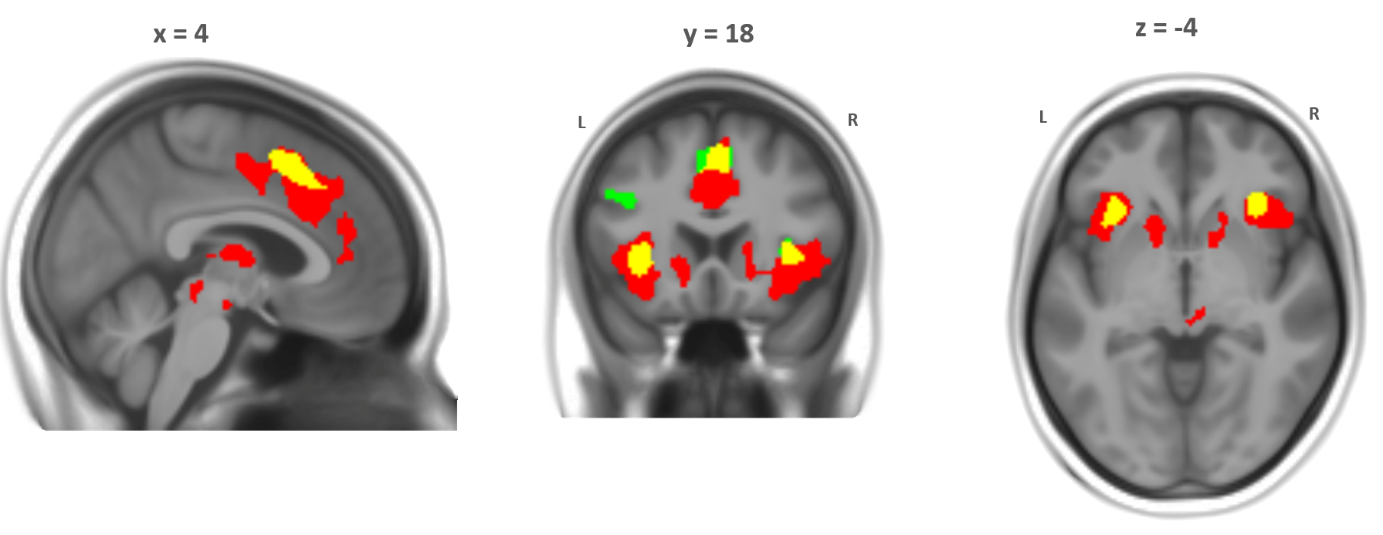
**

**FIGURE S15** Regional overlap (in yellow) of neural correlates of pupil size (in green) and pupil change (in red) (p_FWE.voxel_ < .05 and k > 100)


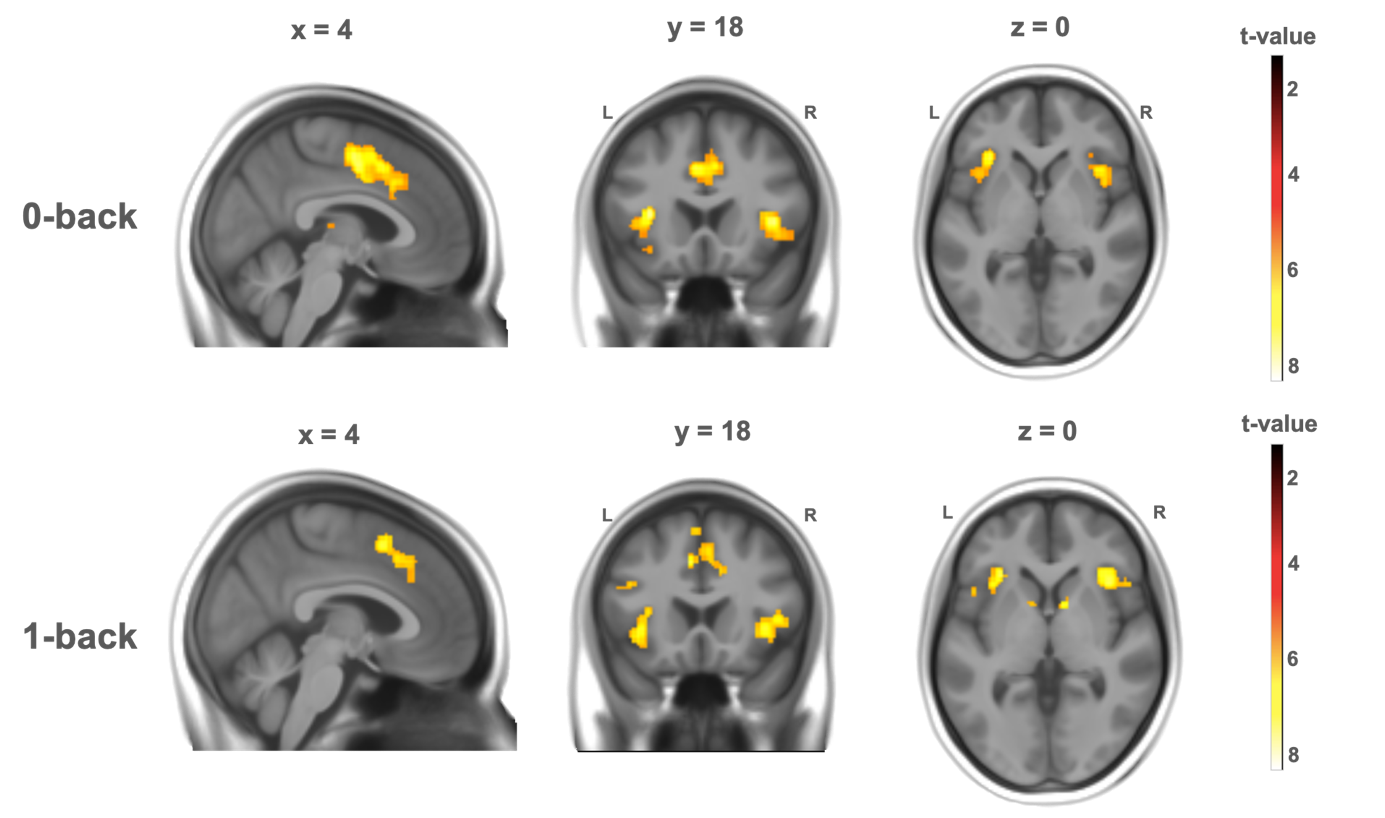


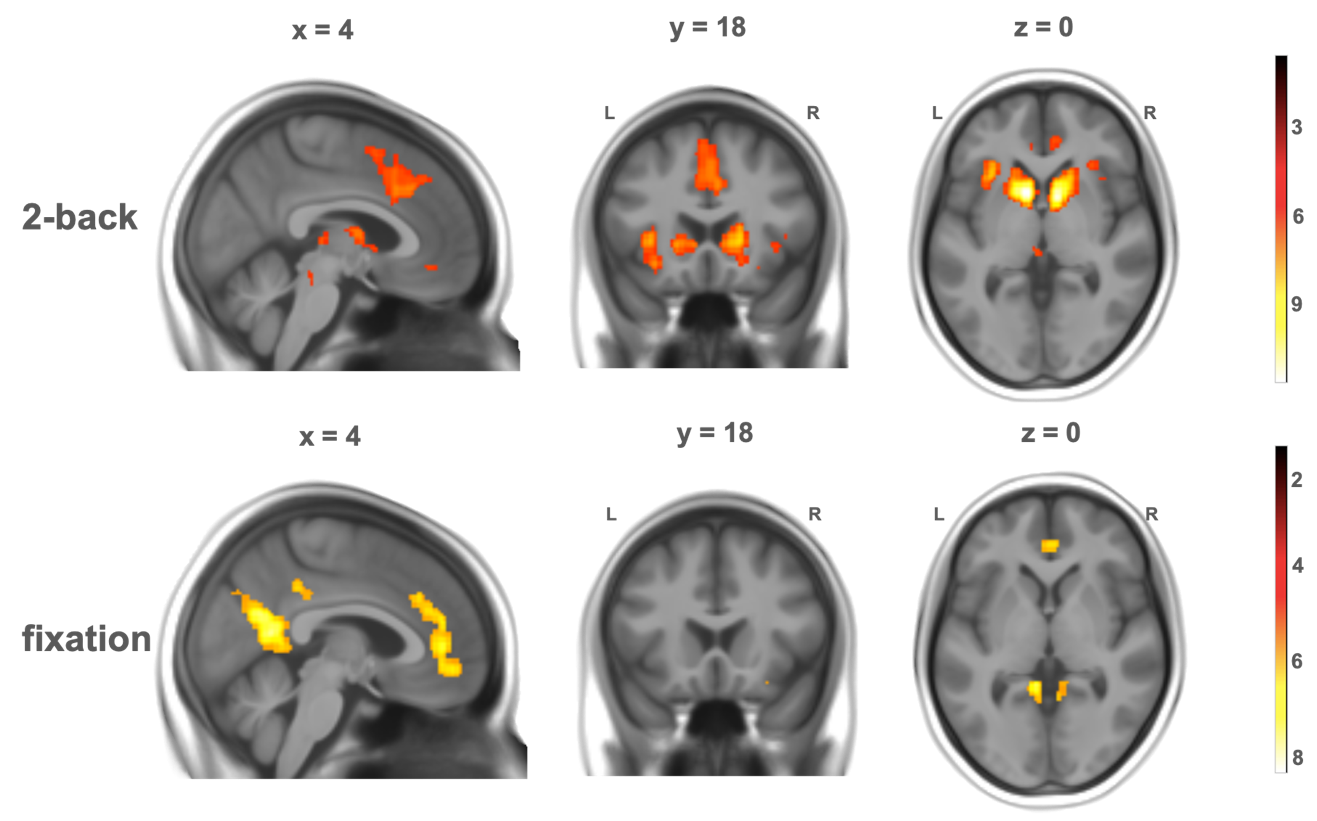


**FIGURE S16** Pupil change neural correlates for each condition separately. (voxel-wise p_FWE_ < .05). L = Left, R = Right


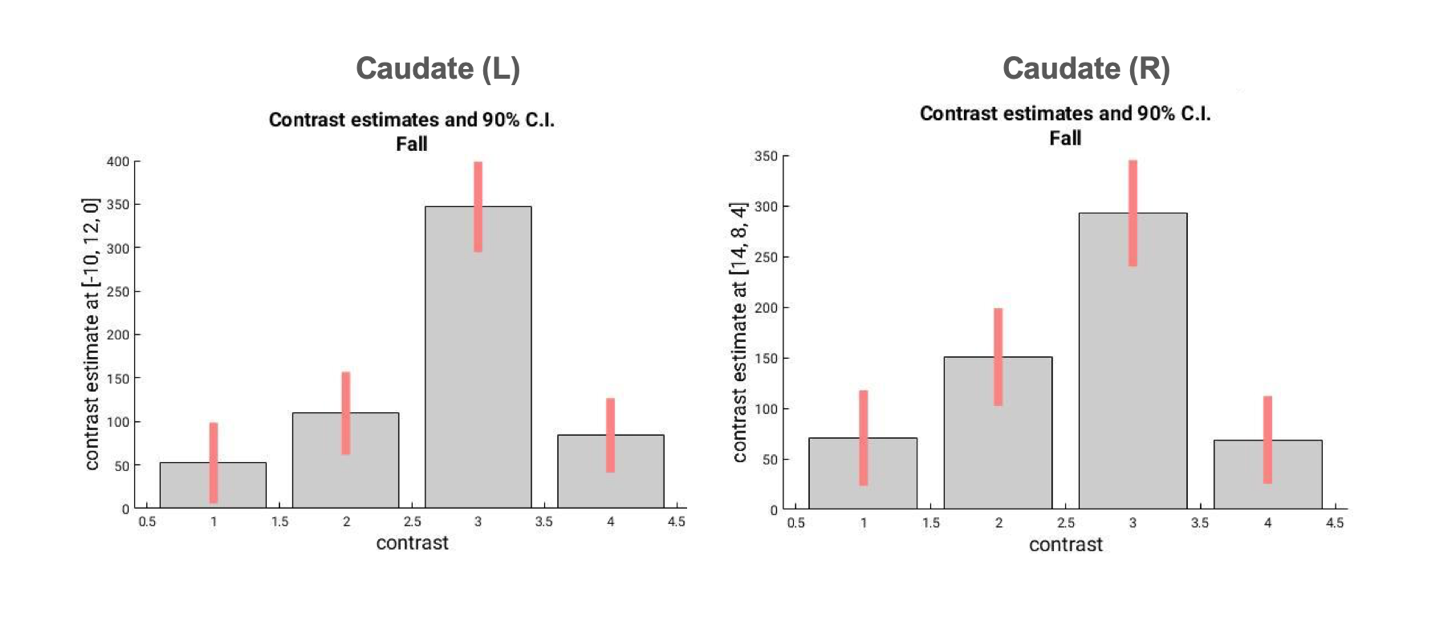


**FIGURE S17** Contrast estimates of the peak voxel (local maximum) of the left and right caudate of the ANOVA main effect. 1 = 0-back, 2 = 1-back, 3 = 2-back, 4 = fixation, L = Left, R = Right

## 3.1 Statistical maps of the main analysis based on frequentist statistics

We have added additional plots of statistical maps of our main analysis using frequentist statistics.


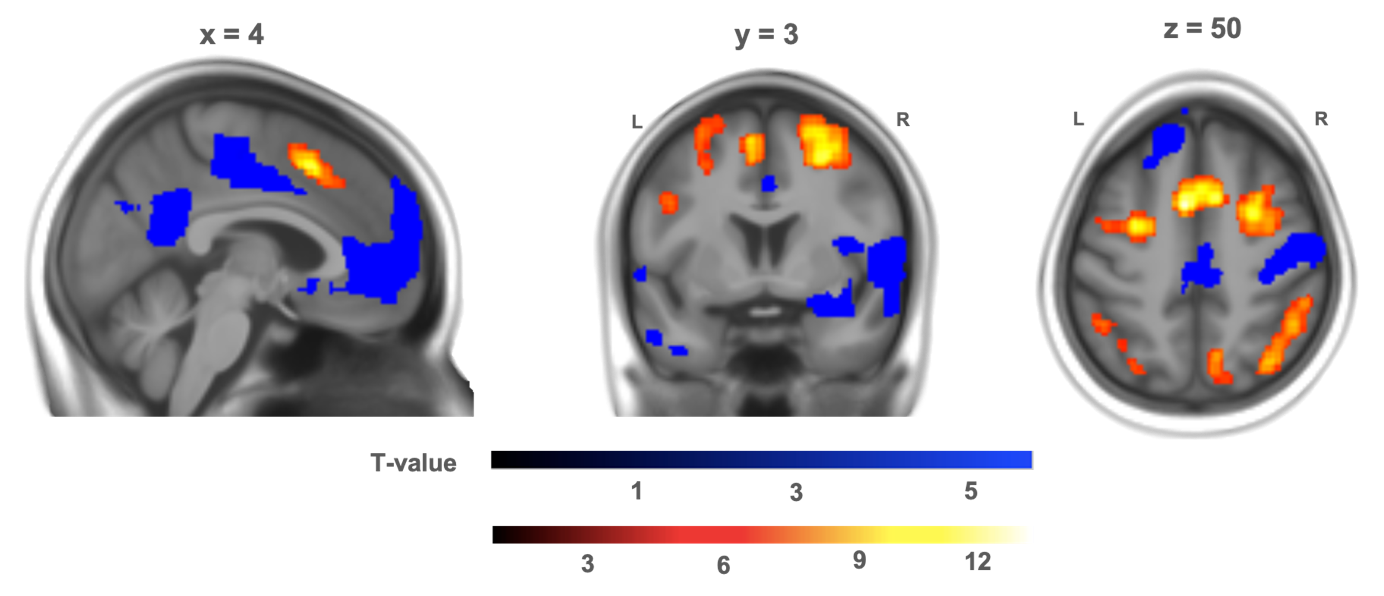


**FIGURE S18** Neural correlates of cognitive load related pupil size between conditions. Hot colors: BOLD activity positively correlated with pupil size. Cold colors: BOLD activity negatively correlated with pupil size. Clusters with p_FWE.voxel_ < .05 and k>100 are shown


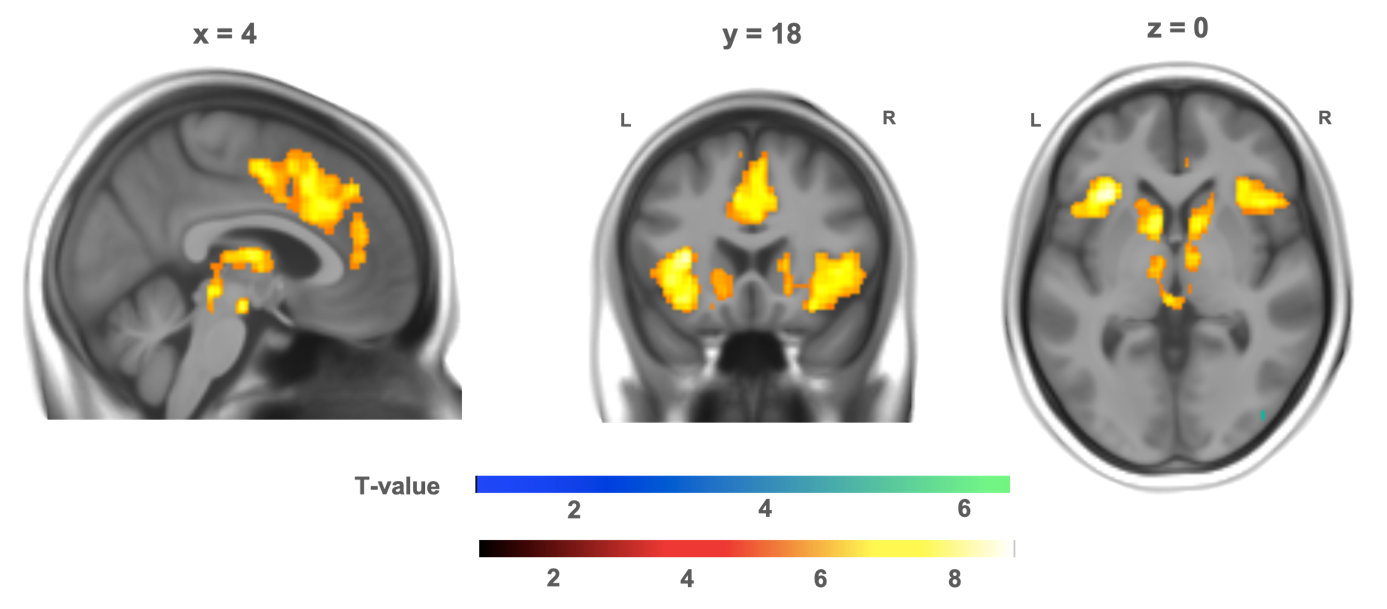


**FIGURE S19** Neural correlates of pupil change within conditions. Hot colors: BOLD activity positively correlated with pupil change. Cold colors: BOLD activity negatively correlated with pupil change. Clusters with p_FWE.voxel_ < .05 and k>100 are shown

# Supplementary tables

**TABLE S1** Post-hoc comparison for RT

|  | | | | | | | | | | | |
| --- | --- | --- | --- | --- | --- | --- | --- | --- | --- | --- | --- |
|  | |  | | Prior Odds | | Posterior Odds | | BF _10, U_ | | error % | |
| 0-back |  | 1-back |  | 0.587 |  | 15.457 |  | 26.314 |  | 1.473e  -7 |  |
|  |  | 2-back |  | 0.587 |  | 3.876e +10 |  | 6.599e +10 |  | 8.313e -14 |  |
| 1-back |  | 2-back |  | 0.587 |  | 1686.479 |  | 2871.087 |  | 5.692e -11 |  |
|  | | | | | | | | | | | |
| Note. U = uncorrected. | | | | | | | | | | | |

**TABLE S2** Post-hoc comparison for accuracy

|  | | | | | | | | | | | |
| --- | --- | --- | --- | --- | --- | --- | --- | --- | --- | --- | --- |
|  | |  | | Prior Odds | | Posterior Odds | | BF _10, U_ | | error % | |
| 0-back |  | 1-back |  | 0.587 |  | 0.324 |  | 0.552 |  | 3.956e -6 |  |
|  |  | 2-back |  | 0.587 |  | 7438.977 |  | 12664.221 |  | 6.520e -7 |  |
| 1-back |  | 2-back |  | 0.587 |  | 237.504 |  | 404.330 |  | 2.217e -9 |  |
|  | | | | | | | | | | | |
| Note. U = uncorrected.  **TABLE S3** Post-hoc comparison for pupil size   \|  \| \| \| \| \| \| \| \| \| \| \| \| \| --- \| --- \| --- \| --- \| --- \| --- \| --- \| --- \| --- \| --- \| --- \| --- \| \|  \| \|  \| \| Prior Odds \| \| Posterior Odds \| \| BF _10, U_ \| \| error % \| \| \| 0-back \|  \| 1-back \|  \| 0.414 \|  \| 158433.583 \|  \| 382492.504 \|  \| 1.383e  -8 \|  \| \|  \|  \| 2-back \|  \| 0.414 \|  \| 1.464e +20 \|  \| 3.534e +20 \|  \| 6.991e -25 \|  \| \|  \|  \| fixation \|  \| 0.414 \|  \| 9.387e  +7 \|  \| 2.266e  +8 \|  \| 1.439e -14 \|  \| \| 1-back \|  \| 2-back \|  \| 0.414 \|  \| 1.394e +13 \|  \| 3.366e +13 \|  \| 4.093e -17 \|  \| \|  \|  \| fixation \|  \| 0.414 \|  \| 4.294e +16 \|  \| 1.037e +17 \|  \| 3.266e -20 \|  \| \| 2-back \|  \| fixation \|  \| 0.414 \|  \| 1.231e +27 \|  \| 2.972e +27 \|  \| 1.887e -32 \|  \| \|  \| \| \| \| \| \| \| \| \| \| \| \| \| Note. U = uncorrected. \| \| \| \| \| \| \| \| \| \| \| \| | | | | | | | | | | | |

**TABLE S4** Pupil size between conditions contrast

| Anatomical cluster location | k | x | y | z |
| --- | --- | --- | --- | --- |
| **Positive contrast** |  |  |  |  |
| Supplementary motor area (L), Supplementary motor area (R) | 496 | -6 | 8 | 48 |
| Insula (R), Inferior frontal gyrus, orbital part (R) | 140 | 34 | 28 | -4 |
| Superior frontal gyrus (R), Middle frontal gyrus (R), Precentral gyrus (R) | 586 | 26 | 6 | 58 |
| Precentral gyrus (L)b, Superior frontal gyrus (L), Middle frontal gyrus (L) | 235 | -28 | -4 | -.48 |
| Cerebellum 6 (R), Cerebellum crus I (R) | 105 | 34 | -64 | -28 |
| Inferior parietal gyrus (L), Middle occipital gyrus (L) | 496 | -46 | -42 | 42 |
| Insula (L), Inferior frontal gyrus, triangular part (L) | 143 | -32 | 24 | -2 |
| Inferior parietal gyrus (R), Angular gyrus (R), Supramarginal gurys (R) | 651 | 48 | -46 | 42 |
| **Negative contrast** |  |  |  |  |
| Medial superior frontal gyrus (L), Anterior cingulate gyrus (L), Medial superior frontal gyrus (R), Medial frontal gyrus (R), orbital part, Medial frontal gyrus (L), orbital part | 3488 | -8 | 52 | 0 |
| Posterior orbitofrontal cortex (R), Orbital inferior frontal gyrus (R), Anterior orbitofrontal cortex (R), Medial orbitofrontal cortex (R) | 239 | 28 | 32 | -14 |
| Superior temporal gyrus (R), Rolandic operculum (R), Supramarginal gyrus (R), Insula (R) | 2154 | 56 | -4 | 8 |
| Postcentral gyrus (R), Precentral gyrus (R) | 1005 | 42 | -18 | 48 |
| Cingulate gyrus mid part (R), Cingulate gyrus mid part (L), Paracentral lobule (L) | 473 | 2 | -14 | 40 |
| Cerebellum crus II (R), Cerebellum crus I (R) | 136 | 32 | -78 | -36 |
| Precuneus (L), Cingulate gyrus posterior part (L), Precuneus (R), Calcarine fissure (L), Cingulate gyrus mid part (L), Posterior cingulate gyrus (R) | 963 | -2 | -48 | 30 |
| Superior temporal gyrus (L), Rolandic operculum (L), Heschl gyrus (L) | 271 | -56 | -4 | -2 |
| Middle occipital lobe (R), Middle temporal lobe (R), Inferior occipital gyrus (R), Inferior temporal gyrus (R) | 344 | 46 | -78 | 0 |
| Angular gyrus (L), Middle occipital gyrus (L), Middle temporal gyrus (L) | 182 | -48 | -70 | 28 |

*Note*. This table refers to Figure 4. Reported clusters survived voxel-wise family-wise error correction for multiple comparisons (p_FWE_ < .001). Only contributions from regions above 5%_cluster_ and minimum number of voxels above 50 are listed. R = right, L = left, [x y z] coordinates are in MNI-space.

**TABLE S5** Pupil change within conditions contrast

| Anatomical cluster location | k | x | y | z |
| --- | --- | --- | --- | --- |
| **Positive contrast** |  |  |  |  |
| Insula (R), Orbital inferior frontal gyrus (R) | 374 | 32 | 26 | -6 |
| Insula (L), Orbital inferior frontal gyrus (L) | 499 | -32 | 24 | -2 |
| Superior frontal gyrus (R), Middle frontal gyrus (R) | 586 | 26 | 6 | 58 |
| Caudate (R) | 153 | 12 | 10 | 6 |
| Thalamus (R), Thalamus (L) | 157 | 0 | -12 | 8 |
| Cingulate gyrus mid part (L), Cingulate gyrus mid part (R), Supplementary motor area (L), Anterior cingulate gyrus (L), Anterior cingulate gyrus (R), Supplementary motor area (R) | 1135 | -8 | 26 | 26 |
| Caudate (L), Pallidum (L), Putamen (L) | 133 | -12 | 4 | 4 |
| Postcentral gyrus (L), Precentral gyrus (L) | 147 | -34 | -28 | 58 |
| **Negative contrast** |  |  |  |  |
| Middle occipital lobe (R) | 32 | 36 | -86 | 6 |

*Note*: Table refers to Figure 5. Reported clusters survived voxel-wise family-wise error correction for multiple comparisons (p_FWE_ < .001). Only contributions from regions above 5%_cluster_ and minimum number of voxels above 50 are listed (the negative contrast is an exception as only one cluster survived the threshold of p_FWE_ <.001). R = right, L = left, k for the cluster size, [x y z] coordinates are in MNI-space.

**TABLE S6** Post-hoc control analysis. Demeaned pupil change within conditions contrast

| Anatomical cluster location | k | x | y | z |
| --- | --- | --- | --- | --- |
| **Positive contrast** |  |  |  |  |
| Caudate (R), Putamen (R) | 181 | 12 | 26 | -6 |
| Insula (L), Orbital inferior frontal gyrus (L) | 477 | -28 | 26 | -6 |
| Middle cingulate gyrus (L), Middle cingulate gyrus (R), Supplementary motor area (L), Anterior cingulate gyrus (R), Anterior cingulate gyrus (L), Supplementary motor area (R) | 1087 | -2 | -10 | 52 |
| Insula (R), Orbital inferior frontal gyrus (R), Inferior frontal gyrus, triangular (R) | 320 | 30 | 20 | -16 |
| Thalamus (R), Thalamus (L) | 154 | 0 | -12 | 8 |
| Caudate (L), Putamen (L), Pallidum (L) | 217 | -12 | 4 | 4 |
| Postcentral gyrus (L), Precentral gyrus (L) | 153 | -34 | -28 | 58 |

*Note*: Table refers to Figure S11. Reported clusters survived voxel-wise family-wise error correction for multiple comparisons (p_FWE_ < .001). Only contributions from regions above 5%_cluster_ and minimum number of voxels above 50 are listed (the negative contrast is an exception as only one cluster survived the threshold of p_FWE_ <.001). R = right, L = left, k for the cluster size, [x y z] coordinates are in MNI-space.

**TABLE S7** Pupil change dependent and independent of conditions contrast (Main effect: voxel-wise p_FWE_ < .05; Conjunction with 4 conditions: uncorrected p < .001; Conjunction with 3 conditions: voxel-wise p_FWE_ < .05)

|  |  |  |  |  |  |
| --- | --- | --- | --- | --- | --- |
| Anatomical cluster location | P_corr_ | k | x | y | z |
| **Main effect** |  |  |  |  |  |
| Caudate (L), Putanem (L) | < .05 | 171 | -10 | 12 | 0 |
| Caudate (R) | < .05 | 227 | 10 | 12 | 0 |
| Cingulate gyrus mid part (L), Cingulate gyrus mid part (R), Posterior cingulate gyrus (L) | < .05 | 111 | 0 | -40 | 36 |
| Inferior frontal gyrus (opercular part) (R), Precentral gyrus (R) | < .05 | 178 | 54 | 8 | 30 |
| Inferior parietal gyrus (L), Postcentral gyrus (L) | < .05 | 281 | -40 | -32 | 46 |
| Middle occipital gyrus (L), Superior occipital gyrus (L) | < .05 | 64 | -22 | -72 | 32 |
| Middle frontal gyrus orbital part (R), Middle frontal gyrus orbital part (L) | < .05 | 55 | 6 | 46 | -10 |
| Calcarine fissure (L), Precuneus (L), Cuneus (L) | < .05 | 63 | -12 | -60 | 16 |
| Supramarginal gyrus (R), Postcentral gyrus (R) | < .05 | 34 | 60 | -22 | 30 |
| **Conjunction (4 conditions)** | P_uncorr_ |  |  |  |  |
| Cingulate gyrus mid part (R), Anterior cingulate gyrus (L), Cingulate gyrus mid part (L), Anterior cingulate gyrus (R), Superior frontal gyrus medial part (L) | <.001 | 584 | 4 | 26 | 32 |
| Insula (R), Inferior frontal gyrus, orbital part (R) | <.001 | 210 | 34 | 24 | -8 |
| Insula (L), Inferior frontal gyrus, orbital part (L) | < .001 | 187 | -32 | 18 | -12 |
| Thalamus (L) | < .001 | 46 | -2 | 20 | 8 |
| **Conjunction (3 conditions)** | P_corr_ |  |  |  |  |
| Insula (L) | < .05 | 156 | -30 | 24 | -6 |
| Insula (R), Inferior frontal gyrus, orbital part (R) | < .05 | 36 | 34 | 24 | -6 |
| Cingulate gyrus, mid part (R), Supplementary motor area (R), Supplementary motor area (L), Medial superior frontal gyrus (L) | < .05 | 93 | 10 | 20 | 36 |

*Note*: This Table refers to Figures S12, S13, and S14. Reported clusters either survived voxel-wise family-wise error correction for multiple comparisons (p_FWE_ < .05) or uncorrected comparison (p_uncorr_ < .001) as indicated. Only contributions from regions above 10%_cluster_ and minimum number of voxels above 30 are listed. R = right; L = left; P_corr_ stands for whole brain corrected cluster p-values and p_uncorr_ stands for uncorrected comparison, k for the cluster size, [x y z] coordinates are in MNI-space.

**TABLE S8** Pupil peak per trial contrast

| Anatomical cluster location | k | x | y | z |
| --- | --- | --- | --- | --- |
| **Positive contrast** |  |  |  |  |
| Precuneus (L), Insula (L), Calcarine fissure (L), Precuneus (R) | 8546 | 0 | -18 | 8 |
| Anterior cingulate gyrus (L), Anterior cingulate gyrus (R), Middle cingulate gyrus (L), Middle cingulate gyrus (R), Supplementary motor area (L), Supplementary motor are (R) | 3307 | 8 | 24 | 28 |
| Insula (R), Inferior frontal gyrus, orbital part (R), Inferior frontal gyrus, triangular part (R), Inferior frontal gyrus, opercular part (R) | 829 | 44 | 18 | -6 |
| Cerebellum 8 (R) | 188 | 16 | -62 | -50 |
| Postcentral gyrus (L), Precentral gyrus (R), Parietal inferior gyrus (L) | 1785 | -52 | -34 | 52 |
| Middle temporal gyrus (L) | 139 | -56 | -22 | -13 |
| Supramarginal gyrus (L), Superior temporal gyrus (L), Rolandic operculum (L), Postcentral gyrus (L) | 630 | -48 | -26 | 22 |
| Insula (L), Rolandic operculum (L) | 135 | -36 | -6 | -18 |
| Middle temporal gyrus (R), Superior temporal gyrus (R) | 181 | 52 | -20 | -8 |
| Superior frontal gyrus 2 (R), Middle frontal gyrus 2 (R) | 171 | 24 | 12 | 46 |
| **Negative contrast** |  |  |  |  |
| Middle occipital gyrus (R), Inferior occipital gyrus (R), Superior occipital gyrus (R), Inferior temporal gyrus (R) | 2178 | 32 | -84 | 10 |
| Middle occipital gyrus (L), Inferior occipital gyrus (L), Inferior temporal gyrus (L) | 1012 | -44 | -58 | -12 |
| Precentral gyrus (R), Postcentral gyrus (R) | 179 | 40 | -16 | 44 |

*Note*: Table refers to Figure 6. Reported clusters survived uncorrected comparison (p_uncorr_ < .001). Only contributions from regions above 5%_cluster_ and minimum number of voxels above 100 are listed. R = right; L = left; k stands for the cluster size, [x y z] coordinates are in MNI-space.

| **TABLE S9** Descriptive statistics on mean pupil size of each block. | | | | | | | | | | | | | |
| --- | --- | --- | --- | --- | --- | --- | --- | --- | --- | --- | --- | --- | --- |
|  | | | | | | | | | | 95% Credible Interval | | | |
|  | | N | | Mean | | SD | | SE | | Lower | | Upper | |
| fix_1 |  | 52 |  | -0.90 |  | 0.40 |  | 0.06 |  | -1.01 |  | -0.79 |  |
| fix_2 |  | 52 |  | -0.83 |  | 0.54 |  | 0.08 |  | -0.98 |  | -0.68 |  |
| 0-back_1 |  | 52 |  | -0.45 |  | 0.49 |  | 0.07 |  | -0.58 |  | -0.31 |  |
| 0-back_2 |  | 52 |  | -0.01 |  | 0.45 |  | 0.06 |  | -0.13 |  | 0.12 |  |
| 1-back_1 |  | 52 |  | 0.01 |  | 0.34 |  | 0.05 |  | -0.09 |  | 0.10 |  |
| 1-back_2 |  | 52 |  | 0.34 |  | 0.45 |  | 0.06 |  | 0.21 |  | 0.46 |  |
| 2-back_1 |  | 52 |  | 0.72 |  | 0.39 |  | 0.05 |  | 0.61 |  | 0.83 |  |
| 2-back_2 |  | 52 |  | 0.92 |  | 0.31 |  | 0.04 |  | 0.84 |  | 1.01 |  |
|  | | | | | | | | | | | | | |

# References

Compton, R. J., Gearinger, D., Wild, H., Rette, D., Heaton, E. C., Histon, S., Thiel, P., & Jaskir, M. (2021). Simultaneous EEG and pupillary evidence for post-error arousal during a speeded performance task. *European Journal of Neuroscience*, *53*(2), 543–555. https://doi.org/10.1111/ejn.14947

Maier, M. E., Ernst, B., & Steinhauser, M. (2019). Error-related pupil dilation is sensitive to the evaluation of different error types. *Biological Psychology*, *141*, 25–34. https://doi.org/10.1016/j.biopsycho.2018.12.013

Rondeel, E. W. M., van Steenbergen, H., Holland, R. W., & van Knippenberg, A. (2015). A closer look at cognitive control: Differences in resource allocation during updating, inhibition and switching as revealed by pupillometry. *Frontiers in Human Neuroscience*, *9*. https://doi.org/10.3389/fnhum.2015.00494
